# Characterising the contribution of auditory and somatosensory inputs to TMS-evoked potentials following stimulation of prefrontal, premotor, and parietal cortex

**DOI:** 10.1162/imag_a_00349

**Published:** 2024-11-01

**Authors:** Mana Biabani, Alex Fornito, Mitchell Goldsworthy, Sarah Thompson, Lynton Graetz, John G. Semmler, George M. Opie, Mark A. Bellgrove, Nigel C. Rogasch

**Affiliations:** School of Psychological Sciences, Turner Institute for Brain and Mental Health, and Monash Biomedical Imaging, Monash University, Victoria, Australia; Behaviour-Brain-Body Research Centre, Justice and Society, University of South Australia, Adelaide, SA, Australia; School of Biomedicine, University of Adelaide, Adelaide, SA, Australia; Hopwood Centre for Neurobiology, Lifelong Health Theme, South Australian Health and Medical Research Institute (SAHMRI), Adelaide, SA, Australia; Discipline of Physiology, School of Biomedicine, University of Adelaide, Adelaide, SA, Australia; Discipline of Psychiatry, Adelaide Medical School, University of Adelaide, Adelaide, SA, Australia

**Keywords:** transcranial magnetic stimulation, electroencephalography, TMS-evoked potentials, sensory, auditory, somatosensory, muscle, dorsolateral prefrontal cortex, premotor cortex, parietal cortex

## Abstract

Transcranial magnetic stimulation (TMS) results in a series of deflections in electroencephalography (EEG) recordings known as a TMS-evoked potential (TEP). However, it remains unclear whether these responses reflect neural activity resulting from transcranial stimulation of the cortex, the sensory experiences of TMS, or a combination of the two. Across three experiments (total n = 135), we recorded EEG activity following TMS to the dorsolateral prefrontal cortex, premotor cortex, and parietal cortex as well as a sensory control condition (stimulation of the shoulder or electrical stimulation of the scalp with a click sound). We found that TEPs showed a stereotypical frontocentral N100/P200 complex following TMS of all cortical sites and control conditions, regardless of TMS intensity or the type of sensory control. In contrast, earlier TEPs (<60 ms) showed site-specific characteristics which were largest at the site of stimulation, although TEP topographies were distorted in a subgroup of individuals due to residual TMS-evoked muscle artefact despite cleaning with independent component analysis. Self-reported sensory experiences differed across sites, with prefrontal stimulation resulting in stronger auditory (click sound perception) and somatosensory input (scalp muscle twitch, discomfort) than premotor or parietal stimulation, a pattern that was reflected in the amplitude of later (N100/P200), but not earlier (<60 ms), TEP peak amplitudes. Later TEPs were also larger in individuals who experienced stronger click sound perception and, to a lesser extent, TMS-evoked scalp muscle twitches. Increasing click sound perception by removing auditory masking increased N100/P200 amplitudes without altering earlier peaks, an effect which was more prominent at sites with more successful masking. Together, these findings suggest that the frontocentral N100/P200 complex primarily represents a generalised sensory response resulting from TMS-related auditory and somatosensory input when present. In contrast, early TEP peaks likely primarily reflect activity resulting from transcranial stimulation of the cortex when artefacts were adequately accounted for. The results have important implications for designing and interpreting TEP studies, especially when comparing TEPs between stimulation sites and participant groups showing differences in sensory experiences following TMS.

## 1 Introduction

Transcranial magnetic stimulation (TMS) is a non-invasive brain stimulation technique that has become increasingly utilised in both experimental and clinical neuroscience. A single TMS pulse induces a set of time-locked deflections in electroencephalographic (EEG) recordings of cortical activity, known as a TMS-evoked EEG potential (TEP) (Ilmoniemi & Kičić, 2010;Rogasch & Fitzgerald, 2013). TEPs reflect neuronal reactivity to TMS, both at the site of stimulation and across the brain, and are sensitive to changes in stimulation parameters (Casarotto et al., 2010), pharmacological manipulation (Premoli et al., 2014), and differences in brain state (Massimini et al., 2005). Furthermore, certain characteristics of TEPs differ across the lifespan and with various neurological and psychiatric disorders (e.g., epilepsy and schizophrenia) (Hui et al., 2019;Tremblay et al., 2019). Despite their promise for understanding human neurophysiology, TEPs are yet to be used as diagnostic markers in clinical contexts as their underlying neurophysiological mechanisms are not well understood (Tremblay et al., 2019). One crucial criterion for TEPs to be considered as indices of cortical excitability is their sensitivity to the stimulation of different neuronal subsets. However, recent studies have shown that stimulation of different cortical areas, with different neuronal compositions, produces TEP components strongly resembling those induced by sensory sham stimulation (which only produced TMS scalp sensations and/or click sound) (Biabani et al., 2019;Conde et al., 2019;Gordon et al., 2018). These findings suggest that TEPs could reflect activity resulting from transcranial stimulation of the cortex, activity resulting from sensory experiences related to the TMS pulse, or a combination of the two.

Given these concerns, a growing body of research has examined the contribution of multisensory potentials to TEPs (Biabani et al., 2019,^2021^;Conde et al., 2019;Gordon et al., 2018,^2021^,2023a;Rocchi et al., 2021). Studies comparing TEPs between stimulation sites have shown that while early latency responses within the first 60 ms following TMS tend to show site-specific topographies and time courses, later responses often converge on a common frontocentral N100 and P200 peak regardless of stimulation site (Freedberg et al., 2020). This suggests that late TEPs are dominated by a stereotypical signal, rather than reflecting site-specific potentials. The spatial and temporal characteristics of late responses are consistent with auditory-evoked potentials (AEPs) resulting from the TMS clicking sound (Nikouline et al., 1999), and somatosensory-evoked potentials (SEPs) resulting from stimulation of cranial/facial nerves and scalp muscles across the scalp (Gordon et al., 2021), which can cause sensations of discomfort or even pain. The multisensory nature of the frontocentral N100/P200 components has led to the term peripherally evoked potentials (PEPs).

While the contribution of AEPs to TEPs has been recognised for several decades (Nikouline et al., 1999), it was generally assumed that the influence of these potentials could be sufficiently minimised by playing continuous masking noise (either white noise or noise adapted from the frequency spectrum of the TMS pulse) through headphones and placing a layer of foam between the scalp (Ilmoniemi et al., 2015). However, recent research on TEPs has shown that these commonly used masking methods are not always sufficient to prevent perception of the coil click, with strong correlations between TEPs and PEPs still often present between 60 and 300 ms after the TMS pulse (Biabani et al., 2019;Gordon et al., 2023b), and in one study as early as 25 ms (Conde et al., 2019). Furthermore, there is ongoing debate as to the relative contribution of AEPs and SEPs to PEPs, with some studies arguing that PEPs are primarily auditory (Ilmoniemi & Kičić, 2010), while others have suggested a combination of both inputs (Conde et al., 2019;Gordon et al., 2021;Rocchi et al., 2021). Given that somatosensory experiences such as discomfort and scalp muscle twitches differ across scalp locations (Meteyard & Holmes, 2018), it is possible that the relative auditory/somatosensory contribution to TEPs could also differ between stimulation locations. Consequently, methods developed for suppressing sensory input in one region may not always translate to others. Understanding the contribution of sensory inputs to TEPs from different stimulation locations is, therefore, paramount for interpreting the neural basis of TMS-evoked EEG activity, and for designing effective methods to improve the reliability of TMS-EEG research across the field.

The aim of the current study was to characterise the contribution of auditory and somatosensory inputs to TEPs following stimulation of three brain regions beyond the commonly studied primary motor cortex: the prefrontal cortex, premotor cortex, and parietal cortex. In Experiment A, we compared TEPs following stimulation of each site to PEPs from TMS over the shoulder, a non-cortical sensory control condition. We also compared self-reported sensory experiences from each site including auditory (loudness of the TMS click) and multi-modal somatosensory (discomfort, pain, scalp muscle twitch) inputs. In Experiment B, we repeated the design of Experiment A in a larger independent cohort and assessed the robustness of these findings to stimulation parameters by using a lower relative stimulation intensity. We then compared individuals with stronger/weaker auditory and somatosensory experiences following stimulation to assess whether the different sensory domains independently altered TEP characteristics. To address limitations with the correlational and between-subject analyses in Experiments A and B, in Experiment C we performed within-subject manipulations of the level of auditory masking for each stimulation site to more directly assess the role of auditory input for TEPs at each site. We also compared TEPs from each stimulation site with PEPs from a more realistic somatosensory control condition (electrical scalp stimulation matched for each site including a concomitant coil click). We hypothesised that the amplitude of common frontocentral N100 and P200 peaks would be modified by varying levels of both auditory and somatosensory input within and between subjects for each site and between sites, whereas earlier site-specific peaks would remain independent of sensory input.

## 2 Methods

### 2.1 Participants

Experiments A and B were a part of a larger, multimodal project aimed at understanding the neural correlates of working memory and how TMS interacts with neural circuits relevant for cognition. This research was conducted at Monash Biomedical Imaging, Monash University, and recruited 29 (17 female, Mean ± SD_Age_: 34 ± 7.25 years) and 94 (58 female, Mean ± SD_Age_: 27.04 ± 7.01 years) healthy adults; respectively. Experiment C recruited 12 healthy individuals (8 female, Mean ± SD_Age_: 23 ± 4.30 years) and was conducted at the Neurophysiology of Human Movement Laboratory, University of Adelaide. The studies were approved by Monash University (Experiments A and B) and University of Adelaide (Experiment C) Human Ethics Committees, and all participants provided their written informed consent prior to participation. Experiments were conducted in accordance with the Declaration of Helsinki and all procedures within the TMS safety guideline of the International Federation of Clinical Neurophysiology were followed (Rossi et al., 2009). During TMS, participants were seated comfortably in an armchair with their eyes open looking straight ahead at a black screen.

### 2.2 EMG

Electromyographic (EMG) activity was measured from the right first dorsal interosseous (FDI) muscle to estimate resting motor threshold. A pair of bipolar surface electrodes (Ag–AgCl with 4 mm active diameter) were placed in a belly-tendon montage with a distance of ~2 cm. The ground electrode was positioned over the midpoint of the middle metacarpal bone in the right hand. EMG signals were sampled at 5 kHz, amplified 1,000 times, band-pass filtered between 10 and 1,000 Hz, and epoched between -200 and 500 ms around the TMS pulse.

### 2.3 EEG

EEG was recorded with a TMS-compatible 62-channel SynAmps^2^EEG system (Neuroscan, Compumedics, Australia) in Experiments A and B and an eego^tm^mylab device (ANT Neuro, Enschede, The Netherlands) in Experiment C. The equipment used included Ag/AgCl-sintered ring electrodes positioned in an elastic cap according to the 10–10 international system (EASYCAP, Germany). The recordings were online referenced to FPz and grounded to AFz. For each individual, the 3D positions of all electrodes were digitised and co-registered to their T1-weighted MRI scan using a neuronavigation system (Brainsight™ 2, Rogue Research Inc., Canada). EEG signals were sampled at 10 kHz, amplified 1,000 times, band-pass filtered between DC and 2 kHz, and recorded by the Curry8 (Neuroscan, Compumedics, Australia; Experiments A and B) and eego (Version 1.9.2, ANT Neuro Corp, The Netherlands; Experiment C) software. Impedance at all channels was kept at below 5 kΩ throughout the session.

### 2.4 TMS

Experiments A and B involved a MagPro X100 Option stimulator (MagVenture, Denmark) with a figure-of-eight coil (C–B60), which was set to deliver single TMS pulses with a biphasic waveform. The direction of the electrical current induced in the underlying cortex was anterior–posterior and then posterior–anterior relative to the coil handle. Experiment C used a Magstim 200^2^stimulator with a figure-of-eight coil (external diameter of each wing 90 mm) delivering monophasic pulses in posterior–anterior direction. The stimulators were controlled by the MATLAB-based MAGIC (MAGnetic stimulator Interface Controller) Toolbox (Saatlou et al., 2018).

Motor hotspot was determined as the position and orientation of the coil that elicited largest motor-evoked potentials (MEPs) in the right FDI muscle at a slightly suprathreshold stimulation intensity. Resting motor threshold (rMT) was defined as the minimum intensity of stimulation over the motor hotspot, with EEG cap on, producing MEPs >50 μV in FDI muscle in at least 5 out of 10 successive trials. Each individual received 4 blocks of 120 trials at the intensities of 120% (Experiment A) or 100% rMT (Experiment B and C), accounting for coil-to-cortex distance (Julkunen et al., 2009;Stokes et al., 2005). The lower TMS intensity utilised in Experiments B and C facilitates the examination of how stimulation intensity affects the impact of PEPs on TEPs. Additionally, the larger sample size provides a more robust dataset, which is particularly beneficial given the low signal-to-noise ratio of TEPs induced by low-intensity TMS. In each block, TMS pulses were delivered with 4–6 seconds intervals (jittered) over one of the targeted cortical sites defined by MNI coordinates (x, y, z), including prefrontal (MNI coordinates: -46, 33, 34), premotor (MNI coordinates: -23, 4, 63), and parietal (MNI coordinates: -26, -69, 63). In addition, shoulder stimulation over the left acromioclavicular joint was added as a control condition to produce PEPs without transcranially activating the cortex. We chose left shoulder stimulation to match the left-sided positioning of the TMS coil in peripersonal space used for scalp stimulation, as both auditory and somatosensory inputs can result in larger evoked potentials in the contralateral hemisphere (Bennett et al., 1987;Picton et al., 1999). Although shoulder stimulation cannot serve as an optimal control condition, our previous study clearly showed that it produces a substantial amount of PEP signals in TEPs (Biabani et al., 2019). The intensity for shoulder stimulation was adjusted subjectively to the perceived intensity of the strongest stimulation required over the scalp.

To account for inter-individual variability in gyrification of the cortex (Thielscher et al., 2011), the anatomical locations of the cortical sites were verified on the T1-weighted MRI for each individual, and coordinates found deep in the sulcus were moved to the adjacent gyrus. The coil was positioned perpendicular to the long axis of the target gyrus to induce the strongest e-field within the area (Thielscher et al., 2011). The MR-based neuronavigation system ensured precise placement and maintenance of TMS coil across trials. To minimise PEPs, a thin layer of foam was attached under the coil and white noise was played through inserted headphones fitted inside disposable foam earplugs worn by participants. To adjust the white noise pressure for each individual, we gradually increased the sound level until the participant was unable to hear the click sound of the TMS coil when discharged at the maximum intensity required across the cortical stimulation conditions or when their upper limit of comfort was reached. The order of the stimulation conditions was counterbalanced for each participant. After each block of stimulation, the participants were asked to score their perception of stimulation on a Numerical Rating Scale (NRS) ranging from 0 to 10 in terms of (i) discomfort (0 = not uncomfortable at all; 10 = highly uncomfortable), (ii) pain (0 = no pain at all; 10 = the worst pain I could tolerate during the experiment), (iii) muscle twitch (0 = no twitches; 10 = very strong cramp), and (iv) click sound (0 = I could not hear the pulses at all; 10 = the pulses were as loud as without white noise).

Within Experiment C, an auditory control condition (TMS without noise masking) and a realistic sham condition were also applied to each of the three cortical sites, resulting in six additional blocks of stimulation. The realistic sham condition was included to account for limitations associated with using shoulder stimulation as a sensory control condition. Unlike shoulder stimulation, the realistic sham condition intended to match somatosensory input to the position of the TMS coil on the scalp using a weak electrical stimulus. For the sham condition, an electrical stimulation (ES) was delivered using a DS7A electric stimulator (Digitimer Ltd., Ft. Lauderdale, FL, United States) through bipolar electrodes (diameter: 8 mm; height: of 3.6 cm) positioned at the site of stimulation over the EEG cap with a distance of 30 mm between the electrodes. Stimulation was delivered as square pulses with the duration of 1,000 μs and a maximum compliance voltage of 400 V, with the timing synchronised to TMS pulses. According to previous studies, these settings produce an ES below the level required to directly activate the cortex (Cohen & Hallett, 1988;Merton & Morton, 1980). For each stimulation site, the intensity of the ES was adjusted to match the sensation induced by real TMS reported by each individual. All other aspects of the ES block were consistent with the real TMS condition except that the TMS coil was tilted at 90° with respect to the scalp to produce the clicks without directly stimulating the cortex.

### 2.5 Data analysis

For Experiment A, EEG data were pre-processed adopting the method described inRogasch et al. (2014,^2017^) with custom scripts written in MATLAB (R2020b; The MathWorks, United States) using functions implemented in EEGLAB (Delorme & Makeig, 2004) and TESA (Rogasch et al., 2017) toolboxes. In brief, the data from all conditions were first concatenated to ensure that cleaning procedures were applied identically across conditions and underwent the following pre-processing steps: epoching around the TMS pulse (-1,000 to 1,000 ms), baseline correction (-500 to -10 ms), TMS pulse artefact removal (-2 to 15 ms) followed by cubic interpolation, downsampling (to 1,000 Hz), visual inspection of trials/channels and artefact removal, removal of TMS-induced muscle and decay artefacts following the FastICA algorithm, a linear interpolation for the missing data, band-pass (1–100 Hz) and band-stop (48–52 Hz) filtering using a zero-phase Butterworth filter (order = 4), and correction of other artefacts (e.g., blinks, channel noise) by applying a second FastICA algorithm (see Supplementary Materials (Table S3) for the detail on the rejected components). Finally, rejected channels were spherically interpolated, and the data were re-referenced to the common average. The scalp EEG cleaning pipeline used in the present study has been described in detail in our recent publication (Biabani et al., 2019) and the scripts can be downloaded from (https://github.com/BMHLab/TEPs-PEPs). The distribution of TMS-induced e-field in the brain was estimated using the SimNIBS software pipeline (www.simnibs.org). First, the individualised head models were reconstructed from T1- and T2-weighted MRIs utilising FreeSurfer (Fischl, 2012) and FSL (Jenkinson et al., 2012) tools. The models consisted of a tetrahedral volume mesh divided into five different tissue types (skin, bone, cerebrospinal fluid, white, and grey matter) with default isotropic conductivity values (Thielscher et al., 2011;Windhoff et al., 2013). Following segmentation and meshing, the finite element method (FEM) was used to estimate e-field values. For each cortical region, individualised e-field maps were generated using the stimulation intensity, time-varying current in the coil (dI/dt(A/s)), MNI coordinates of the coil centre, and the orientation of the coil in space. Both source and e-field maps of each individual were transformed to the default FSAverage surface template parcellated with Desikan–Killiany atlas (Desikan et al., 2006) for group analysis. Global mean field power (GMFP) was calculated using the method introduced byLehmann & Skrandies (1980),

$$GMFP\left(t\right)=\sqrt{\frac{\left[\sum_{i}^{k}{\left({\text{V}}_{i}\left(t\right)-{\text{V}}_{mean}\left(t\right)\right)}^{2}\right]}{\text{k}}},$$ (1)

where t is time, K is the number of channels, Vi is the voltage at channel i, and Vmean is the mean voltage across all channels.

For Experiments B and C, scalp EEG data were pre-processed using the same method as adopted in Experiment A. However, the ES condition in Experiment C had one additional pre-processing step for removing the decay artefact using the method employed byConde et al. (2019). In this method, the exponential modelA*exp(B*x)+C*exp(D*x)was fitted to the raw data where x was the time between 5 and 500 ms post-stimulus. The parameters A, B, C, and D were estimated using fit*()*function in MATLAB with exp2 as argument. The best fit was then subtracted from each trial and each channel to remove the decay.

All statistical tests were performed in MATLAB using custom scripts. Normality of data distribution was tested using Shapiro–Wilk normality tests. Comparisons across conditions were conducted using one-way repeated measure ANOVA (rmANOVA) or Friedman tests depending on the distribution, followed by false discovery rate (FDR)-corrected pairwise comparisons of paired samples. Pairwise comparisons between conditions utilised paired-sample t-tests or Wilcoxon signed-rank tests based on the distribution of the data. Spatiotemporal correlations between the potentials among the stimulation conditions were examined using Spearman’s correlation coefficients. The correlation tests were first performed at the individual level and then the coefficients (ρ) were transformed to z values employing Fisher’s transformation for group-level statistics (Dunn & Clark, 1969;Hopkins, 1998). The z-scores were tested against zero using one-sample permutation tests with 10,000 iterations and t_max_correction to evaluate the statistical significance of correlations (Blair & Karniski, 1993). For illustration purposes, we transformed z back to ρ values.

## 3 Results

### 3.1 Experiment A

**Fig. 1.**
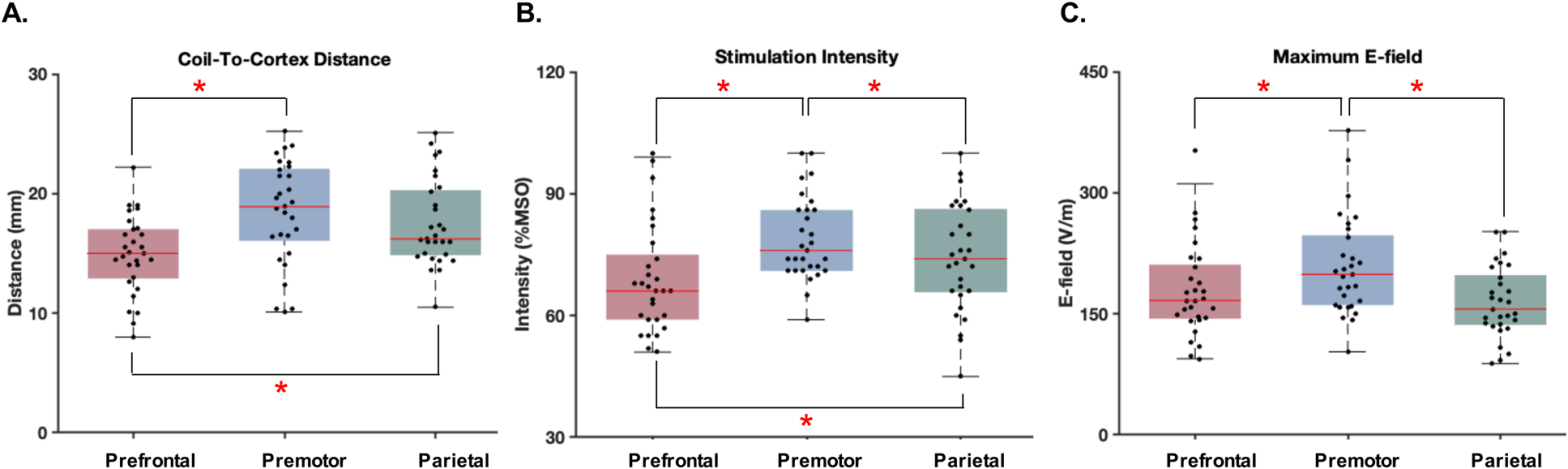
Anatomical measures and stimulation parameters. A) Distance from the centre of the coil to the stimulation target on the cortical surface, B) stimulation intensity as a % of maximum stimulator output (MSO), and C) maximum estimated E-field evoked by TMS in the cortex calculated from a finite element model. Each dot in the box and whisker plots represents the value for each individual. The shaded boxes highlight the 25th to 75th centiles of the values and the red horizontal lines within the boxes show the median of the values. *Indicates significant difference between the stimulation conditions (FDR-corrected*p*< 0.05).

All the measurements were well tolerated by all participants without any serious adverse effects.Figure 1provides the individual values of the anatomical measures and stimulation parameters for Experiment A. Coil-to-cortex distance was significantly different across stimulation sites (Mauchly’s*W*= 0.96,*p*= 0.59; one-way rmANOVA:*F*(2, 56) = 15.02,*p*< 0.001) with the smallest distance at prefrontal cortex and largest distance at premotor cortex (paired-sample t-tests FDR-corrected; prefrontal-premotor,*p*= 0.002; prefrontal-parietal,*p*= 0.002; premotor-parietal,*p*= 0.19). Accordingly, prefrontal cortex received the weakest and premotor cortex received the strongest stimulation intensity (Mauchly’s*W*= 0.84,*p*= 0.10; one-way rmANOVA:*F*(2, 56) = 13.96,*p*< 0.001; paired-sample t-tests FDR-corrected; prefrontal-premotor,*p*= 0.003; prefrontal-parietal,*p*= 0.02; premotor-parietal,*p*= 0.01). Despite adjusting the stimulation intensity for coil-to-cortex distance, the strength of the generated e-field at the level of cortex was different across stimulation sites. As the assumption of sphericity was not met (Mauchly’s*W*= 0.74,*p*= 0.02), Greenhouse–Geisser-corrected values were considered (one-way rmANOVA:*F*(1.58, 44.31) = 8.95,*p*= 0.001; paired-sample t-tests FDR-corrected; prefrontal-premotor,*p*= 0.002; prefrontal-parietal,*p*= 0.23; premotor-parietal,*p*= 0.002), suggesting the commonly used coil-to-cortex correction method is insufficient for matching TMS-evoked e-fields between sites (Fig. 1).

#### 3.1.1 Self-reported sensory experiences of TMS pulses

**Fig. 2.**
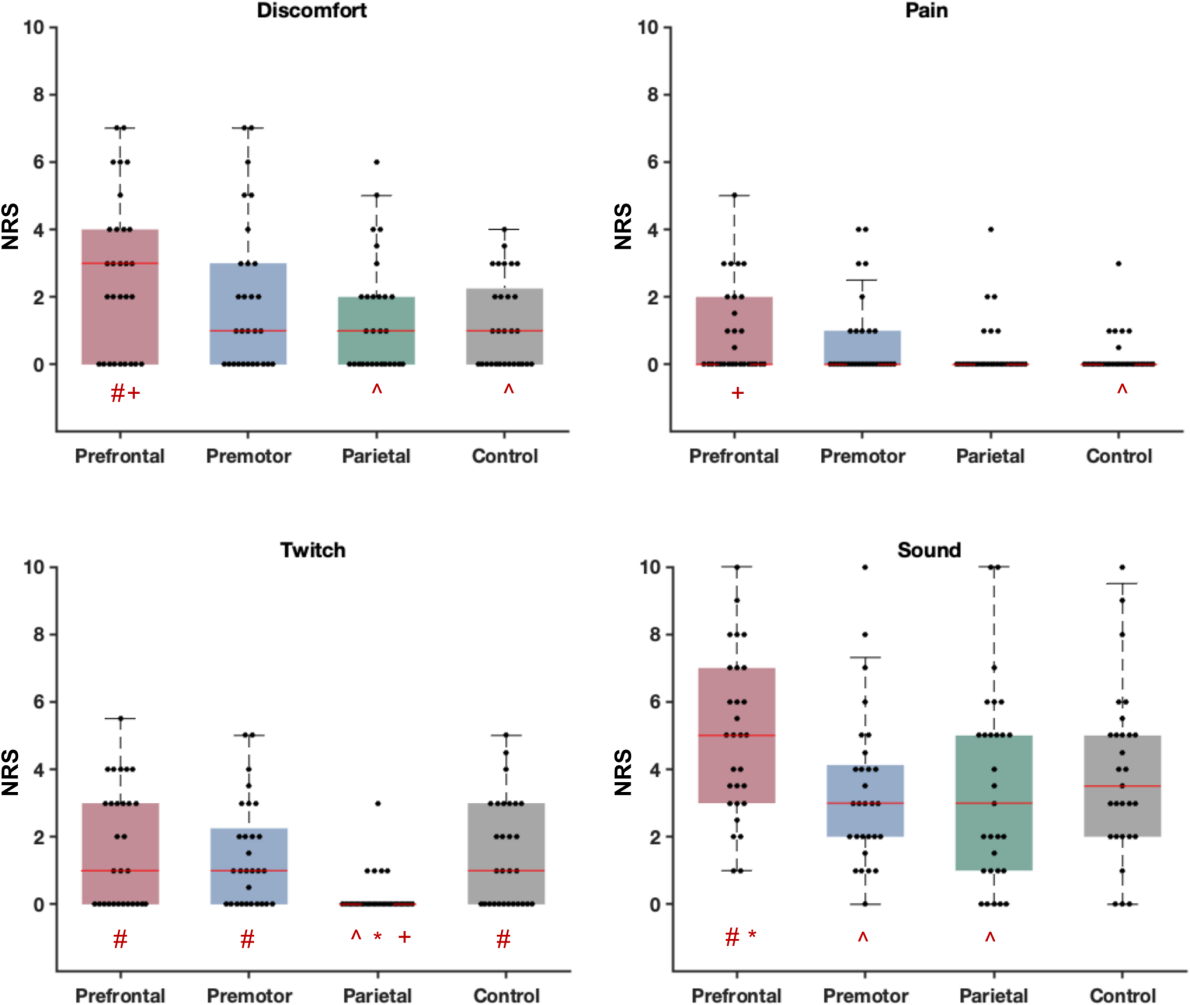
Experiment A: Self-reported perception of discomfort, pain, muscle twitch, and click sound caused by real and control stimulation conditions. Each dot in the box and whisker plots represents the Numerical Rating Scale (NRS) score for each individual. The shaded boxes highlight the 25th to 75th centiles of the values and the red horizontal lines within the boxes show the median of the values. ^, *, #, and + indicate significant difference (FDR-corrected*p*< 0.05) with prefrontal, premotor, parietal, and control, respectively.

Figure 2shows comparisons between sensory experiences following TMS pulses across the different stimulated regions in Experiment A. All participants reported an NRS greater than 0 in at least one perception/condition. According to Shapiro–Wilk normality tests, NRS values were not normally distributed in all conditions (*p*> 0.05). Therefore, we used Friedman tests to compare each sensation across the stimulation conditions, followed by FDR-corrected Wilcoxon signed-rank tests for pairwise comparisons. Comparing somatic sensations across cortical sites, parietal cortex stimulation resulted in the lowest ratings of muscle twitch (chi square = 15.58,*df*= 3,*p*= 0.001; prefrontal-premotor,*p*= 0.62; prefrontal-parietal,*p*= 0.003; prefrontal-control,*p*= 0.78; premotor-parietal,*p*= 0.004; premotor-control,*p*= 0.83; parietal-control,*p*= 0.004), pain (chi square = 7.98,*df*= 3,*p*= 0.04; prefrontal-premotor,*p*= 0.54; prefrontal-parietal,*p*= 0.11; prefrontal-control,*p*= 0.03; premotor-parietal,*p*= 0.27; premotor-control,*p*= 0.12; parietal-control,*p*= 0.54), and discomfort (chi square = 8.79,*df*= 3,*p*= 0.03; prefrontal-premotor,*p*= 0.27; prefrontal-parietal,*p*= 0.04; prefrontal-control,*p*= 0.03; premotor-parietal,*p*= 0.27; premotor-control,*p*= 0.27; parietal-control,*p*= 0.69). Of note, nearly all conditions reported some perception of the TMS click sound, even though white noise was played through in-ear headphones in an attempt to mask the click sound. Despite having the lowest stimulation intensity, TMS over prefrontal cortex resulted in the strongest perception of the click sound compared with other sites (chi square = 9.47,*df*= 3,*p*= 0.02; prefrontal-premotor,*p*= 0.01; prefrontal-parietal,*p*= 0.04; prefrontal-control = 0.18; premotor-parietal,*p*= 0.87; premotor-control,*p*= 0.44; parietal-control,*p*= 0.66). These findings show that the sensory experiences differ between stimulation sites across the scalp.

#### 3.1.2 Comparisons of TMS-evoked potentials following stimulation of different sites

**Fig. 3.**
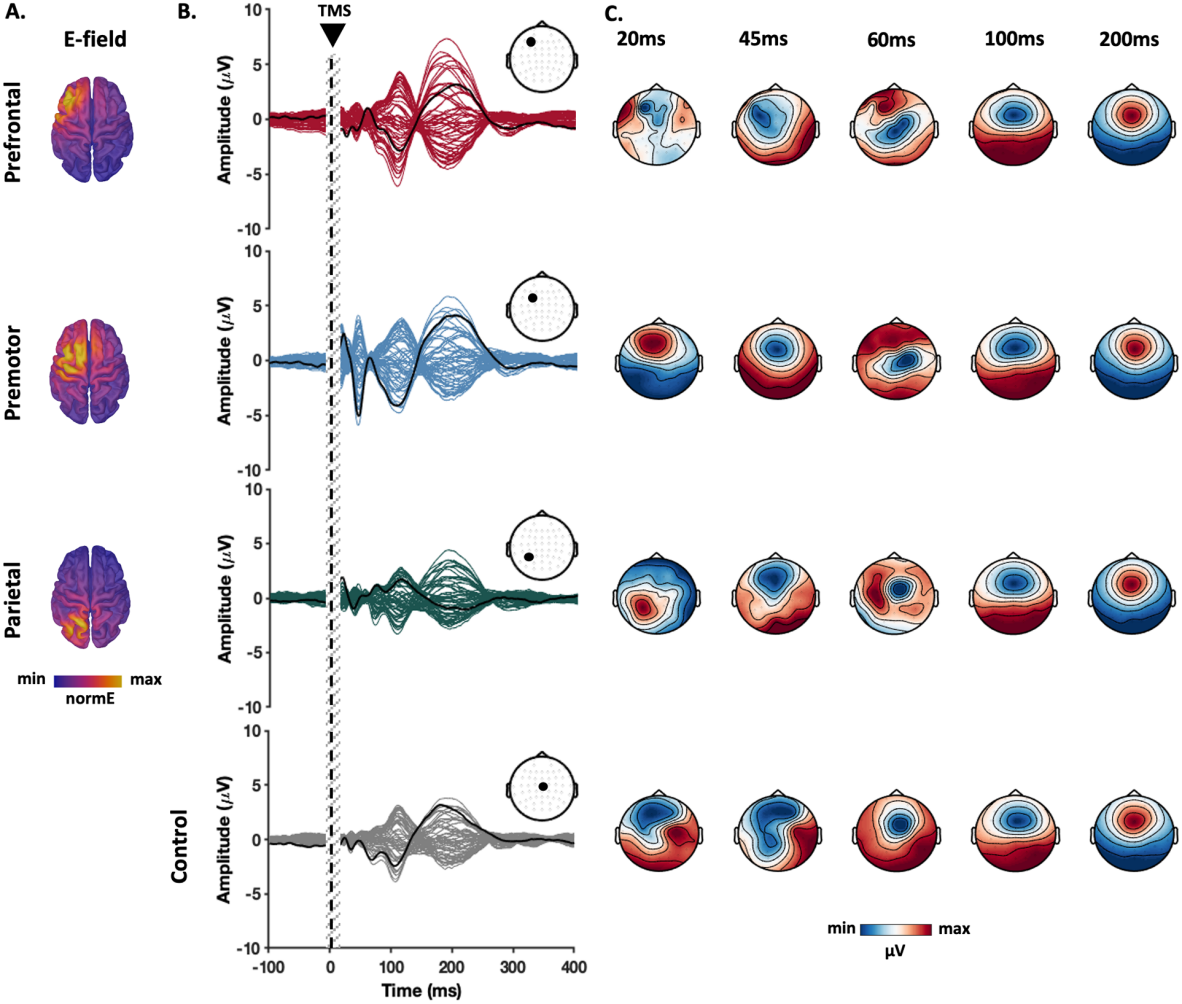
TMS-evoked potential and e-field distributions. (A) Group average of the estimated e-fields in each stimulation condition. (B) Butterfly plots indicate TEPs recorded by each electrode averaged across individuals and the thick black line represents the potentials recorded at the targeted site (prefrontal: F3; premotor: FC1, parietal: P3 and shoulder: CZ). The corresponding electrode positions are marked on the scalp map displayed above. The vertical grey bars demonstrate the time window of the potentials not considered for the analysis. (C) Topographical maps depict distribution of the potentials across the scalp around the time points that TEP peaks appeared. Note that topoplots following prefrontal cortex stimulation at 20 and 45 ms show similarities to TMS-evoked scalp muscle activity despite cleaning with ICA.

After the data cleaning process for Experiment A, the number of remaining trials (mean ± SD) in different conditions was as follows: prefrontal: 103.24 (13.27), premotor: 101.68 (11.01), parietal: 103.06 (11.30), and control: 104.10 (10.62).Figure 3illustrates the spatiotemporal distribution of TMS-evoked e-fields and TEPs following stimulation of each site. The TMS-evoked e-fields show clear spatial separation of the stimulated cortical regions when targeting the different cortical sites (Fig. 3A). Together, the butterfly plots (Fig. 3B) and topoplots (Fig. 3C) suggest differences in TEPs between stimulation sites within the first 60 ms following stimulation, which converge on a similar pattern between 60 and 300 ms between sites, although the amplitudes appear to differ. Of note, potential residual TMS-evoked scalp muscle activity is evident in the topoplots following prefrontal cortex stimulation at 20 and 45 ms post-stimulation.

**Fig. 4.**
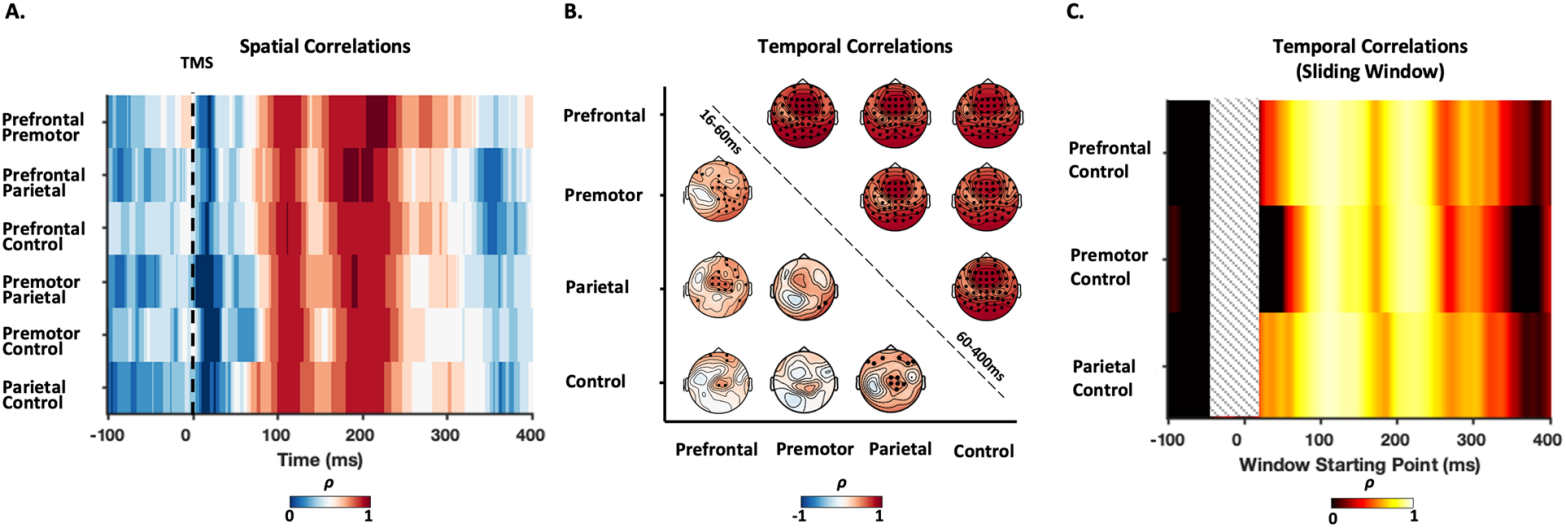
Pairwise spatial and temporal correlations between TEPs from different conditions. (A) Temporal changes in Spearman’s correlation between the topographies of each two conditions. (B) Temporal distribution of the Spearman’s correlation coefficients (ρ) between the potentials recorded by the same electrodes in each two conditions at two different time windows: early (16–60 ms; lower triangle) and late (60–400 ms; upper triangle). The channels highlighted in black demonstrated significant correlations (*p_corrected_*< 0.05) between the two conditions. (C) Temporal correlations between TEPs following cortical stimulation and the control condition from the FCz electrode. Correlations were calculated using a fixed length of 50 ms and advanced in 1 ms steps across the data (i.e., a sliding window approach).

To compare similarities between TEPs following stimulation of different sites, we performed correlation analyses in both the spatial and temporal domains. Pairwise spatial correlations (i.e., comparing the similarities of topoplots at each point in time) showed weak correlations between topographies within the first 60 ms post-stimulation, with strong correlations present between 60 and 300 ms poststimulus that peaked around 100 and 200 ms across all comparisons (Fig. 4A). Pairwise temporal correlations comparing the shape of TEPs within electrodes between sites were performed across two time windows: early (15–60 ms) and late (60–400 ms). We chose these time windows based on our previous work, which showed strong correlations between prefrontal and parietal TEPs starting at about 60 ms and extending beyond 300 ms (Rogasch et al., 2020). The early time window revealed weak associations for most electrode comparisons, with the exception of a cluster of central electrodes which showed moderate strength correlations for most comparisons. In contrast, the late time window showed strong correlations between sites across all electrodes which peaked in frontocentral electrodes (Fig. 4B), suggesting very similar TEP shapes between both active and control conditions. To ensure the differences in temporal correlation between early and late time windows were not due to differences in window length, we performed a sliding-window temporal correlation analysis with a fixed window length (50 ms) between the cortical stimulation sites and the sensory control (shoulder stimulation) using a frontocentral electrode (FCz). This approach has the added benefit of assessing how temporal correlations between TEPs evolve over time. For each site, temporal correlations with the sensory control condition became stronger with windows starting at ~65 ms and lasting until ~250 ms. As with spatial correlations, clear peaks were evident at 100 and 200 ms (Fig. 4C). The strong spatial and temporal correlations from 60 ms between both active and control stimulation sites (e.g., the shoulder) suggest a common underlying neural source between conditions which is not dependent on direct cortical stimulation and is most likely the result of sensory input. The low spatial and temporal correlations before 60 ms between TEPs could reflect differences in the underlying neural activity resulting from stimulation of different cortical sites, differences in residual artefact profiles between sites, or a combination of the two.

**Fig. 5.**
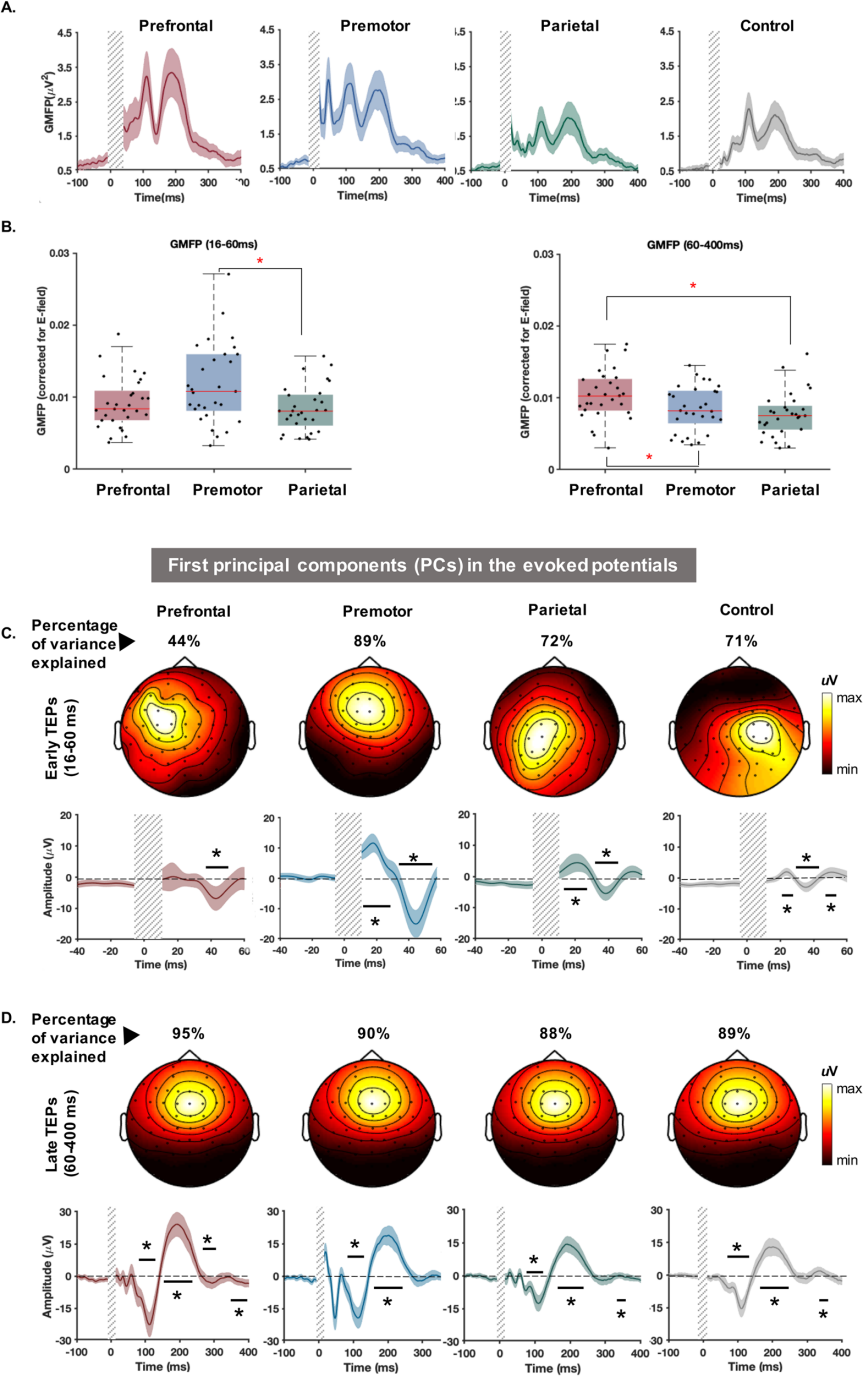
GMFP and the first principal component of the potentials recorded at each stimulation condition in Experiment A. (A) Fluctuations in GMFP following TMS. (B) Comparison of GMFP across conditions at early and late time windows. Each dot in the box and whisker plots represents the value for each individual. The shaded boxes highlight the 25th to 75th centiles of the values and the red horizontal lines within the boxes show the median of the values. *Indicates significant difference between the stimulation conditions (FDR-corrected*p*< 0.05). (C) The dominant PCs identified in early TEPs recorded between 16 and 60 ms. (D) The dominant PCs identified in late TEPs recorded between 60 and 400 ms. The values above the scalp maps indicate the percentage of variance explained by the depicted component for each condition. The line graphs illustrate the changes of PCs amplitude over time. The thick lines represent the group-averaged signal and the shaded areas show 95% CIs of the individual values. The vertical grey bars demonstrate the time window of the potentials not considered for the analysis. The horizontal lines with * indicate when the TEPs significantly deviate from baseline (corrected*p*< 0.05).

To assess differences in the magnitude of TEPs between stimulation sites, we compared GMFP between sites across early (15–60 ms) and late (60–300 ms) time windows. To account for variations in the peak e-field, we normalised the GMFP by dividing it by the peak e-field value (Fig. 5A, B). Comparisons of GMFP showed a significant difference between TEPs across conditions for early (Mauchly’s*W*= 0.82,*p*= 0.07; one-way rmANOVA:*F*(2, 56) = 5.73,*p*= 0.005) and late time windows (Mauchly’s*W*= 0.97,*p*= 0.47; one-way rmANOVA:*F*(2, 56) = 9.90,*p*< 0.001). Post hoc pairwise comparisons, followed by FDR correction, revealed that the premotor cortex had significantly larger GMFP than the parietal cortex (*p*= 0.009) in the earlier time window. In the late time window, prefrontal cortex GMFP was larger than both premotor (*p*= 0.003) and parietal (*p*= 0.0009) cortices showing a similar pattern to differences in sensory perceptions (Fig. 2).

To further understand the similarities and differences between TEPs across the different time windows, we performed principal component analysis (PCA) on the early (16–60 ms) and late (60–400 ms) TEPs separately and identified the components most representative of the signals (i.e., explaining the maximum spatial variance) in the group-averaged data. TEPs from each individual were then weighted according to the detected PC maps to find the dominant temporal patterns. In line with the results from the correlation analyses, PCs from the earlier time window demonstrated site-specific topographies, which tended to peak close to the site of stimulation (electrodes showing maxima for each condition; prefrontal = FC3; premotor = FCz; parietal = CP1). Moreover, the time series from PCs characterising the dominant early TEPs from each stimulated site showed differing peaks, including an N40 (peak = 42 ms) for prefrontal cortex, a P20 (peak = 19 ms) and N45 (peak = 46 ms) for premotor cortex, and a P20 (peak = 20 ms) and N35 (peak = 37 ms) for parietal cortex (Fig. 5B). The peak-to-peak amplitude of the components was largest following premotor cortex stimulation: the condition with the strongest e-field (Fig. 1C;Fig. S1). Of note, the first PC following prefrontal cortex stimulation had lower explained variance than other sites, suggesting another contributing source to early TEPs from this site, possibly residual contamination by TMS-evoked scalp muscle activity (seeFig. 3). In contrast, PCA of late TEPs revealed that at least 88% of the variance in the potentials wase explained by similar frontocentrally distributed potentials for all sites (electrode showing maxima = FCz in all sites), with prominent N100 (maxima range = 111–115 ms across sites) and P200 peaks (maxima range = 191–202 ms across sites) (Fig. 5C). The amplitude of the frontocentral N100 and P200 peaks followed a similar pattern to sensory experiences, and were largest following prefrontal cortex stimulation, and smallest following parietal cortex stimulation (Fig. S1). Together, these findings suggest that early time periods are dominated by site-specific TEPs which are consistent with activity resulting from stimulation of the targeted cortical region. In contrast, late time periods are dominated by TEPs with a common spatiotemporal pattern across stimulation sites including from non-cortical areas (i.e., the shoulder) which likely result from sensory input associated with the TMS pulse.

#### 3.1.3 Assessing residual artefacts in frontal TEPs

Although early latency TEPs showed differing spatiotemporal patterns between sites, it is possible that these differences were related to residual artefacts which were insufficiently cleaned using ICA, as opposed to differences in TMS-evoked neural activity. For example, TMS-evoked scalp muscle activity results in large compound muscle action potentials within the first 10–15 ms following TMS which are orders of magnitude larger than neural-related TEPs, and typically larger/more frequent following stimulation of lateral sites like the dorsolateral prefrontal cortex (seeFig. 2for self-report scalp muscle twitches) (Mutanen et al., 2013;Rogasch et al., 2013). While we removed the data around the TMS pulse capturing the peaks of muscle activity (-2 to 15 ms), the tail can last for tens to hundreds of ms which we attempted to minimise using ICA. However, the large amplitude and time-locked nature of muscle artefacts can violate several ICA assumptions, resulting in either undercleaning, where not all components representing muscle activity are removed leaving residual artefacts in the signal, or overcleaning where muscle and neural signals are mixed in the same components and removed from the final TEP (Atti et al., 2024;Hernandez-Pavon et al., 2012;Metsomaa et al., 2014). To test these possibilities, we assessed uncleaned, epoched TEPs and identified conditions/individuals in which TMS-evoked scalp muscle activity was visible between 5 and 15 ms (for details seeFig. S2). We chose to begin the window at 5 ms as in some participants, the amplifier was offline for up to 5 ms post-TMS. Furthermore, ringing artefacts from the TMS pulse artefact are present for up to 5 ms at the sampling rates used in this study (Rogasch et al., 2013;Veniero et al., 2009). We note that this experimental arrangement precludes us from detecting scalp muscle artefacts within the first few ms following TMS which are theoretically possible, although we are not aware of any reports of such early scalp muscle artefacts in the literature. We then compared the final cleaned TEPs between individuals with muscle activity and no muscle activity following prefrontal and premotor cortex stimulation. We reasoned that if cleaning with ICA resulted in under/over correction of residual muscle activity, then the cleaned group-level TEPs from the two subgroups (muscle/no muscle) should differ.

**Fig. 6.**
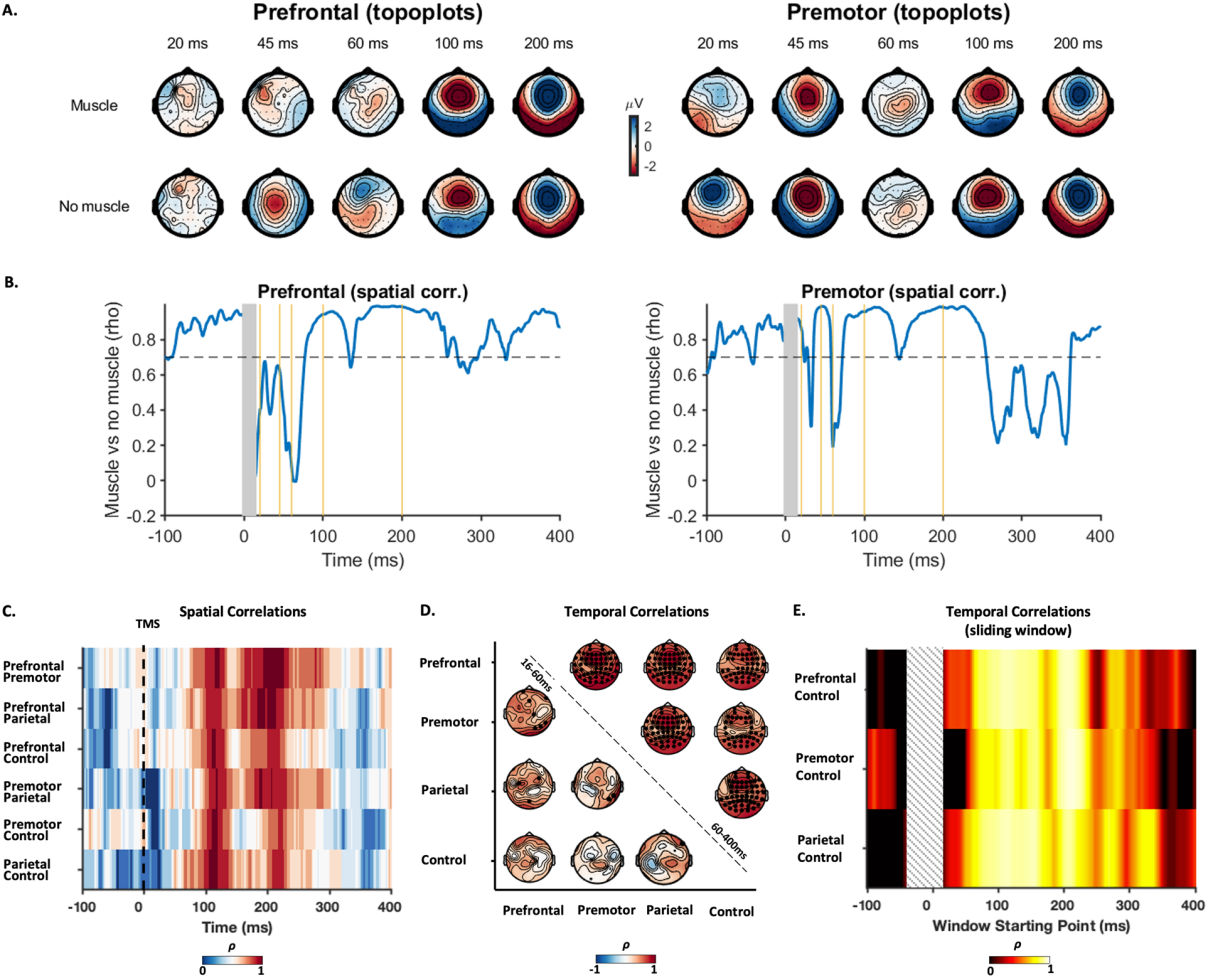
Comparisons of TEPs in individuals with and without TMS-evoked scalp muscle activity immediately following the TMS pulse. (A) Topoplots showing the spatial distribution of cleaned TEP peaks in individuals with and without TMS-evoked scalp muscle activity. Topographies representative of residual scalp muscle activity are evident following prefrontal cortex stimulation despite cleaning with ICA. (B) Spatial correlations between cleaned group mean TEPs in individuals with and without TMS-evoked scalp muscle activity. A clear drop in correlation values between groups is evident in the first 80 ms following prefrontal, but to a lesser extent in premotor cortex stimulation. (C–E) Re-evaluation of spatial and temporal correlations between TEPs in a subgroup of individuals with no TMS-evoked scalp muscle activity in any stimulation condition (n = 9). A similar pattern of results was observed to those inFigure 4, suggesting residual scalp muscle activity was not driving the low correlation values between conditions over early time points.

TMS-evoked muscle activity was visible in 62.1% of individuals following prefrontal, 34.5% following premotor, and 0% following parietal cortex stimulation, with prefrontal stimulation resulting in larger amplitude muscle potentials compared with premotor stimulation. Uncleaned GMFP data showed a clear tail of muscle activity lasting ~100 ms following prefrontal cortex stimulation (Fig. S3). Cleaned GMFP amplitudes were similar in amplitude between subgroups for prefrontal and premotor stimulation sites; however, the PC1 topography showed clear evidence of residual muscle activity following prefrontal cortex stimulation (Fig. S3). This spatial pattern was evident in topoplots covering the first 60 ms and resulted in a drop in spatial correlations of TEPs between the muscle and no muscle subgroups (Fig. 6A, B). In contrast, GMFP and PC1 topographies/time series and correlations were similar between subgroups following premotor cortex stimulation and for both stimulation sites over later time points covering the N100/P200 complex (Fig. 6;Fig. S3). These findings suggest that residual TMS-evoked muscle activity did distort the spatiotemporal pattern of early TEPs in a subgroup of participants following prefrontal, but not premotor cortex stimulation, and only for the early time period. To ensure the low spatial and temporal correlations in early TEPs between sites observed inFigure 4was not being driven by residual artefacts, we reran the analysis on a subset of individuals with no observable TMS-evoked scalp muscle activity following stimulation of any site (n = 9). The pattern of correlations was similar following re-analysis (Fig. 6C–E), suggesting the site specificity of TEPs during the early time period was not simply the result of differences in residual scalp muscle activity.

### 3.2 Experiment B

The first aim of Experiment B was to assess whether the findings from Experiment A replicated in a larger independent dataset (n = 94) with different stimulation settings, in this case a lower relative TMS intensity (100% of rMT). TEPs are still present at lower TMS intensities (as low as 60% RMT;Komssi et al., 2004) and threshold or subthreshold intensities are commonly used in TMS-EEG experiments to help minimise certain sensory inputs (Premoli et al., 2014). Furthermore, the larger sample size in Experiment B increased statistical power to detect subtle differences between TEPs from different sites and different sensory profiles. Stimulation parameters and the level of pulse perceptions for Experiment B are presented in the Supplementary Materials (Table S1;Fig. S4). As illustrated inFigure S5, the distribution of TEPs in Experiment B closely resembled those observed in Experiment A. All of the main findings replicated between studies, including moderate temporal correlations between conditions in central electrodes during the early time period (Fig. S5C), strong temporal and spatial correlations between conditions in the late time period (Fig. S5D), site-specific PC1s for the early time period (Fig. S6A), and common frontocentral PC1s with N100 and P200 peaks for the late time period (Fig. S6B). Note that e-field distribution was not estimated in Experiment B; however, the GMFP results resembled those from Experiment A before correction, with higher GMFP amplitudes for the late time period corresponding to N100/P200 peaks in prefrontal compared with parietal cortex. Together, these findings show that the pattern of site-specific early TEPs and site-general late TEPs replicates for a lower TMS intensity, providing further evidence that the late TEPs likely reflect sensory input regardless of stimulation intensity.

**Fig. 7.**
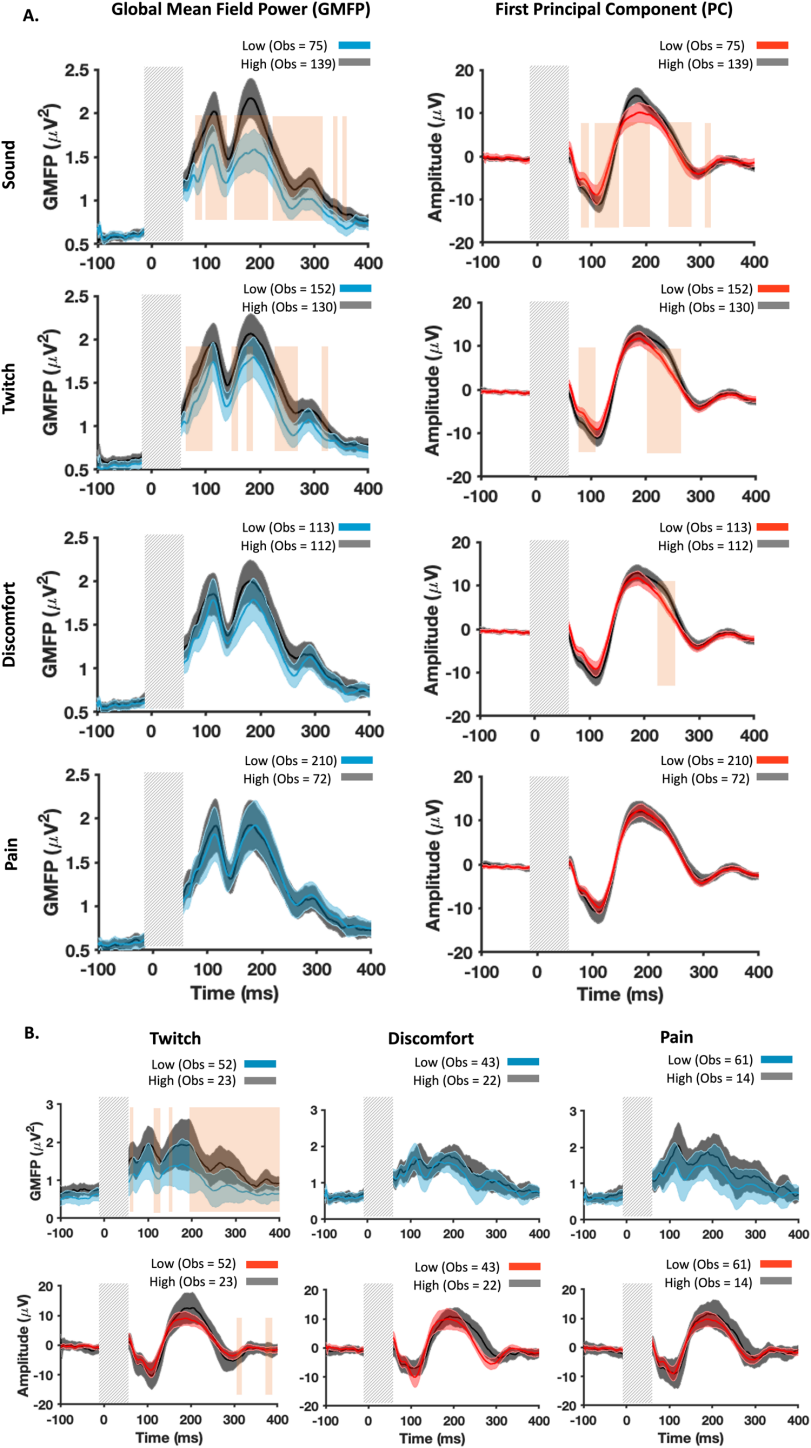
Experiment B: Comparisons of the late potentials between the individuals with high and low levels of sensory perceptions. (A) Differences in the GMFP (left column) and the amplitude of the first PCs (right column) between the individuals who reported a low versus high level of sensory perception. (B) Changes in the GMFP (upper row) and amplitude of the first PCs (lower row) from the subset of participants who perceived a low level of click sound (NRS < 2). Potentials from all stimulation conditions are pooled into each group. The thick lines represent the group-averaged signal and the shaded areas show 95% CIs of the individual values. The vertical grey bars demonstrate the time window of the potentials not considered for the analysis. The orange vertical boxes cover the windows of time showing significant differences between groups (FDR-corrected*p*< 0.05). Note: The sample sizes differ across the perception groups because participants reporting median perception values were not included. “Obs” indicates the number of observations (pooled across participants and stimulation sites) included in each comparison.

The between-site comparisons from Experiments A and B both show that later TEPs are larger in the prefrontal than in the parietal cortex, a pattern which is also observed in self-report sensations of discomfort, scalp muscle twitch strength, and click loudness. To investigate the contribution of different sources of sensory input to the amplitude of late TEPs, we took advantage of the large sample size in Experiment B and stratified the groups based on the presence/absence of each sensory perception. Stratification was based on low perception (NRS < median) versus high level of perception (NRS > median) for the four examined sensations including sound (median NRS = 2), twitch (median NRS = 0), discomfort (median NRS = 1), and pain (median NRS = 0). When median was 0, all the individuals with no perception were assigned to the low NRS group. All participants reported an NRS greater than 0 in at least one perception/condition. As there was no evidence for site specificity in the contribution of later potentials in our findings, we combined the data from all the three real stimulation conditions to further improve the power of analysis (both GMFP and the first PC). This is particularly important as median split stratification can lead to loss of information about individual variability, reducing effect sizes and experimental power (Iacobucci et al., 2015). The point-by-point Wilcoxon signed-rank tests, followed by FDR correction for testing multiple time points, showed that late TEPs (>~60 ms) were mostly larger in the individuals with stronger perceptions (Fig. 7A). The difference in TEPs was prominent between the groups separated by sound perception across a wide range of time windows including the N100 and P200 peaks (Fig. 7A). However, for muscle twitch and discomfort, differences were mainly limited to earlier (~80–90 ms) and later (~220–280 ms) time periods. The results did not show a significant impact of pain perception on the magnitude of TEPs. This finding suggests that although both auditory and somatosensory inputs alter the amplitude of frontocentral signals, auditory inputs make a larger contribution to differences in the N100/P200 peaks. The low impact of somatosensory potentials observed in our findings could have potentially been driven by the confounding effect of different levels of noise masking across groups. As there were no correlations between NRS of sound and other sensations (all FDR-corrected*p*> 0.05), each somatosensory group could have a mixture of both high and low levels of auditory perceptions. To make sure the sound masking was equivalent across the somatosensory comparisons, we selected a subset of participants reporting low NRS (<2) for sound perception, and stratified the groups based on the presence/absence of each somatosensory perception. The results from Wilcoxon signed-rank tests across time points did not reveal significant differences between groups with the exception of muscle twitch particularly after ~250 ms post-TMS, suggesting that SEPs from the evaluated perceptions do make a contribution to TEPs, but not necessarily at the N100/P200 peaks (Fig. 7B). Distributions of NRS for each sensory perception are presented in the Supplementary Materials (Fig. S7). Additionally, we assessed the robustness of these findings by employing an alternative stratification approach, dividing participants into groups based on NRS scores of 0 and greater than 0 for each condition. As illustrated inFigure S8, the results remained consistent, highlighting sound as having the most significant impact on TEP amplitudes among the evaluated perceptions.

### 3.3 Experiment C

**Fig. 8.**
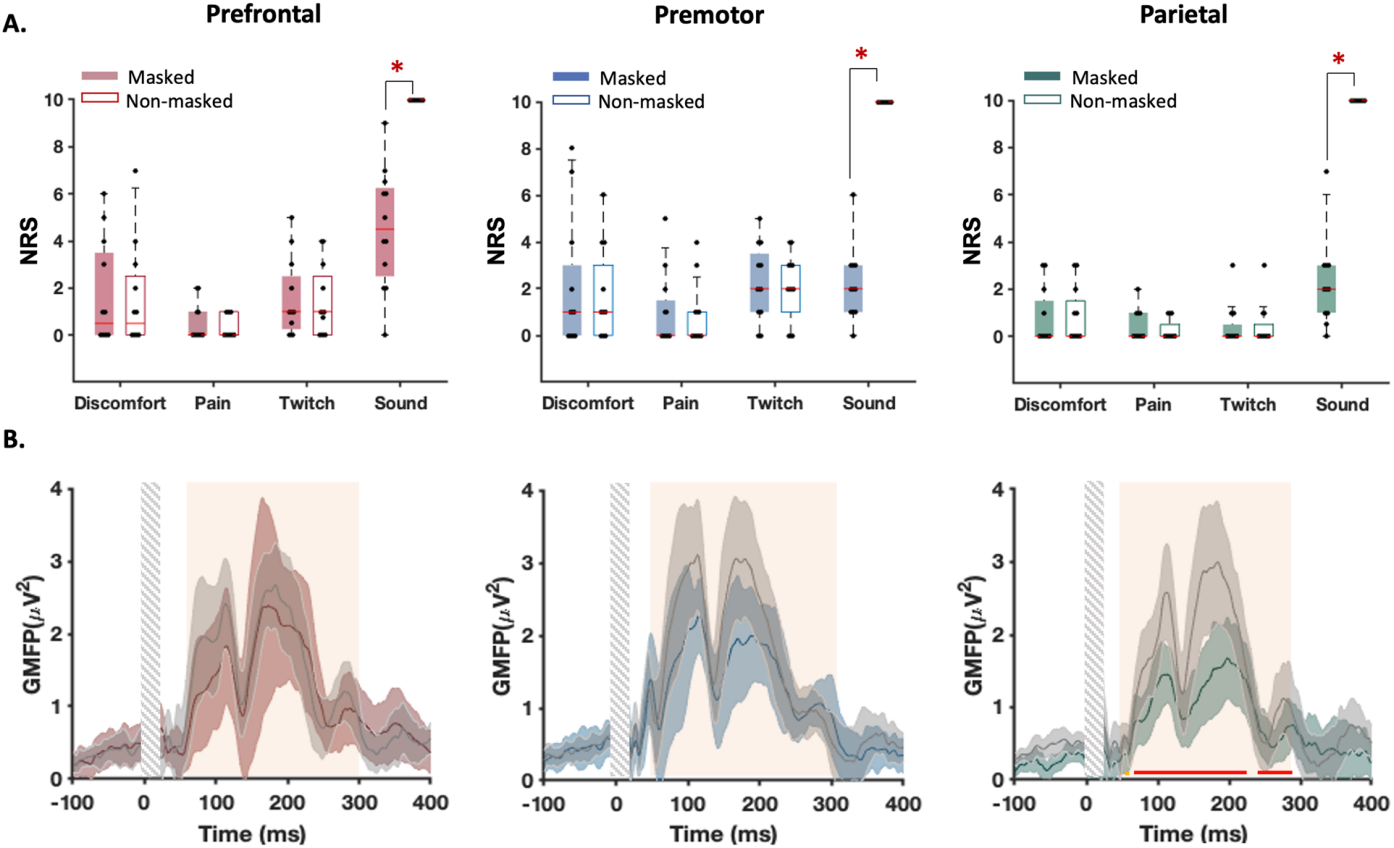
Experiment C: Sensory perceptions and TEPs from real (TMS with noise masking) and auditory control (TMS without noise masking) stimulation conditions. (A) Self-reported perception of discomfort, pain, muscle twitch, and click sound caused by each stimulation condition. All participants reported an NRS greater than 0 in at least one perception/condition. (A) Each dot in the box and whisker plots represents the data for each individual. The shaded boxes highlight the 25th to 75th centiles of the values and the red horizontal lines within the boxes show the median of the values. *Indicates significant difference between the stimulation conditions (FDR-corrected*p*< 0.05). (B) Group-averaged GMFP evoked by TMS with and without noise masking. The shaded areas show 95% CIs of the values and the vertical grey bars demonstrate the time window that was not considered for the analysis. The orange vertical boxes highlight the time windows associated with late potentials where statistical comparisons were made (60–300 ms). The horizontal lines positioned above the zero line represent intervals of significant point-by-point differences (FDR-corrected*p*< 0.05).

Findings from Experiments A and B show that perception of the TMS click sound contributes to the N100/P200 peak amplitudes, and both click perception and N100/P200 amplitude differ between stimulation sites. To assess the role of TMS sound perception more directly in N100/P200 peak amplitude across stimulation sites, we performed an additional experiment which included similar conditions to Experiment B, and a condition in which the auditory masking was removed altogether for each stimulation site while other sensations (i.e., discomfort, pain, and muscle twitch) were kept at the same level (Fig. 8A). We reasoned that if late, but not early, TEPs contained AEPs, then increasing the amount of auditory input perceived by the participant should differentially alter the amplitude of early versus late TEPs. Furthermore, given that auditory masking was more successful in some sites (e.g., parietal cortex) compared with others (e.g., prefrontal cortex), we reasoned that late latency TEP peaks should change more when altering the level of auditory masking at parietal compared with prefrontal sites. TEPs from Experiment C mostly replicated those from Experiment B (Table S2; Figs. S9–S12). Similar to Experiments A and B, across all cortical sites, auditory masking was least successful at reducing perception of the click sound for prefrontal cortex stimulation (Wilcoxon signed-rank test; FDR-corrected*p*: prefrontal-premotor = 0.04; prefrontal-parietal = 0.03; premotor-parietal = 0.87;Fig. 8A).Figure 8Billustrates the changes in GMFP following the removal of auditory masking over time. As shown, the early potentials (<60 ms) remained largely unchanged between conditions with and without noise masking, while the late signals exhibited substantial changes over time (60–300 ms). Wilcoxon signed-rank tests revealed an increase in the average of late potentials at both the parietal (*p*< 0.0001) and premotor stimulation sites (*p*= 0.04) when noise masking was removed. Point-by-point comparisons within this time frame (60–300 ms) showed significant variations at the parietal site for most time points (FDR-corrected*p*< 0.05) (Fig. 8B). We also found point-by-point differences at the premotor site during the 150–200 and 200–260 ms intervals (all*p*< 0.05), which did not remain significant after FDR correction (all*p*> 0.05). No significant changes in late potentials were detected at the prefrontal stimulation site, both in averaged analysis (*p*= 0.32) and in point-by-point comparisons (all*p*> 0.05). Together, these findings suggest (1) auditory-evoked potentials contribute to late, but not early, latency TEPs and (2) differences in the amplitude of late TEPs between stimulation sites correspond closely with variations in the success of auditory masking between sites.

**Fig. 9.**
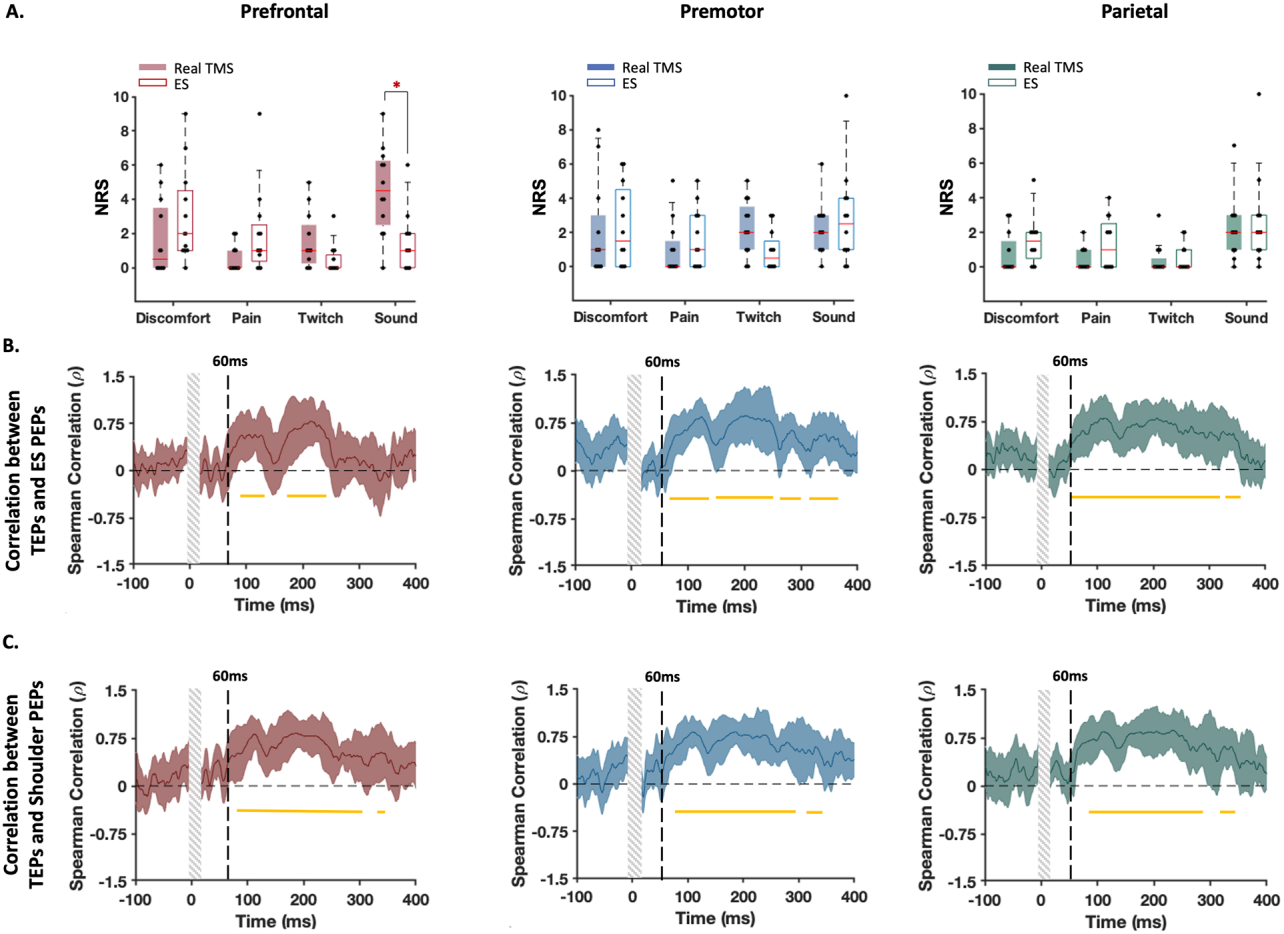
Experiment C: Sensory perceptions and TEPs from real TMS and somatosensory control (electrical stimulation) conditions. (A) Self-reported perception of discomfort, pain, muscle twitch, and click sound caused by each stimulation condition. Each dot in the box and whisker plots represents the Numerical Rating Scale (NRS) score for each individual. The shaded boxes highlight the 25th to 75th centiles of the values and the red horizontal lines within the boxes show the median of the values. *Indicates significant difference between the stimulation conditions (FDR-corrected*p*< 0.05). (B, C) Changes in correlations between the GMFP of the responses to real and sham stimulations over time. The horizontal lines below the zero line indicate the windows of significant correlations. The thick line represents the group-averaged values. The shaded areas show 95% CIs of the values and the vertical grey bars demonstrate the time window that was not considered for the analysis.

An additional limitation from Experiments A and B is that the sensory control condition was performed by stimulating the shoulder, which does not control for spatial differences in somatosensory input when stimulating different cortical/scalp locations. To address this limitation, we performed a further site-specific control condition which involved applying a weak electrical stimulation to the same scalp location while the TMS coil was held away from the scalp, thereby preventing active cortical stimulation by the TMS pulse. The perceived sensations were not different between the two conditions in all stimulation sites except that in the prefrontal cortex, the click sound was louder in real stimulation (*p*= 0.009) (Fig. 9A). Spatial correlations showed significant associations between the real and control TEPs from ~60 ms, maximising around 100 and 200 ms, in accordance with the N100/P200 complex of AEPs (Fig. 9B). As depicted inFigure 9B and C, this correlation pattern closely resembles that between TEPs and PEPs from shoulder stimulation, further suggesting a minimal contribution from somatosensory inputs potentially triggered by scalp stimulation to early TEPs. Similar findings were observed when only analysing individuals without visible TMS-evoked scalp muscle activity in the raw EEG data (Fig. S13).

## 4 Discussion

In the present study, we examined the EEG responses to TMS following stimulation of prefrontal, premotor, and parietal cortex and evaluated their associations with the EEG responses to sensory stimulation. We aimed to identify the features in TEPs that were sensitive to auditory and somatosensory inputs and those which were not. There were four main findings. First, we replicated the strong temporal and spatial correlations between TEPs from different sites and sensory-evoked potentials between 60 and 300 ms, which peaked around the frontocentral N100 and P200 potentials, suggesting these late peaks primarily reflect sensory potentials. We showed that these relationships are replicated for different stimulation intensities (100% and 120% of scalp-to-cortex adjusted RMT) and for different types of sensory stimulation (shoulder stimulation and site-specific electrical stimulation of the scalp with a coil spacer). Second, we found that sensory experiences differed following stimulation of different sites, with prefrontal cortex stimulation resulting in more discomfort, stronger scalp muscle twitches, and louder perception of the coil click (despite white noise masking) compared with the other sites. We also found that the N100/P200 peaks were larger in the prefrontal cortex than in the parietal cortex, showing a similar pattern to sensory experience. Third, we observed that late TEPs were stronger in individuals who perceived louder click sounds and larger scalp muscle twitches. Fourth, we found that removing noise-masking increased N100/P200 amplitude to differing extents across different stimulation sites, with both click perception and TEP amplitudes more strongly modulated at parietal than at prefrontal cortex. Importantly, across all experiments, the early latency TEPs (<60 ms) showed site-specific topographies and time courses and were not altered following any sensory comparison or manipulation, suggesting that these peaks most likely reflect neural activity resulting from direct stimulation of the cortex by TMS. However, we also found that early TEPs can be distorted by residual muscle activity despite cleaning with ICA, an issue that was largest for the prefrontal cortex. Together, these findings provide strong evidence that later TEP peaks are sensitive to auditory and somatosensory experiences of TMS, which can differ between stimulation sites across the scalp, whereas earlier peaks likely primarily reflect activity initiated by transcranial stimulation of the cortex when TMS-evoked artefacts are adequately accounted for.

A growing number of studies have shown strong correlations between frontocentral N100 and P200 PEPs resulting from sensory sham conditions and TEPs following stimulation of multiple sites, including motor, prefrontal, premotor, parietal, and visual cortex (Biabani et al., 2019;Chowdhury et al., 2022;Conde et al., 2019;Gordon et al., 2021;Herring et al., 2015;Rocchi et al., 2021) and cerebellum (Fernandez et al., 2021). The present findings replicate this relationship in prefrontal, premotor, and parietal cortex across three independent datasets (total n = 135), providing further evidence that PEPs contribute to TEPs regardless of stimulation site, stimulation intensity, and the method of sensory stimulation used for comparison (e.g., shoulder vs. electrical scalp stimulation). While we were not able to replicate the strong spatial correlations between PEPs and prefrontal/parietal TEPs at ~25 ms and ~40 ms as reported byConde et al. (2019), we did see a moderate temporal correlation in central electrodes between conditions during early (16–60 ms) time windows. However, we also observed site-specific TEPs with independent spatial and temporal profiles which dominated the early time windows, suggesting some early peaks are primarily reflective of transcranial-evoked activity. We replicated previous studies showing the strength of self-reported sensory experiences such as discomfort, scalp muscle twitches, and perceived coil clicks (regardless of masking) differed across TMS scalp locations (Meteyard & Holmes, 2018), and were larger/stronger following stimulation of prefrontal compared with parietal cortex. We extended these findings by showing the stimulation sites with stronger sensory experience were accompanied by larger N100/P200 peak amplitudes, a relationship which was not observed in early latency TEPs. These findings provide an important insight suggesting that the contribution of PEPs to TEPs may differ across stimulation sites depending on differing sensory experiences.

An important unresolved question is the relative contribution of auditory and somatosensory experience to PEPs following TMS. Some studies have argued PEPs are entirely auditory in origin (Ilmoniemi & Kičić, 2010;Paus et al., 2001), whereas others have suggested both auditory and somatosensory inputs contribute to PEPs (Conde et al., 2019;Gordon et al., 2021;Rocchi et al., 2021). We used between-subject comparisons to isolate the impact of different sensory perceptions including click sound, muscle twitch, pain, and discomfort on the magnitude of the later responses to TMS. We found that TEPs were significantly larger in individuals with stronger perception of the TMS click sound and TMS-evoked scalp muscle twitches. In a follow-up experiment, we found that removing auditory masking increased frontocentral N100/P200 peak amplitudes within participants without altering early latency TEPs, replicating similar findings from the motor cortex (Rocchi et al., 2021;Ter Braack et al., 2015). Furthermore, the modulation of TEP amplitudes was stronger in the sites with more effective auditory masking. Together, these results suggest that auditory input is the major contributor to PEPs in TEPs, although somatosensation also makes a contribution. Notably, the central cortical distribution of cortical activity observed at the N100/P200 (Fig. 3) implies that these peaks are attributable to multiple sources, rather than exclusively reflecting auditory potentials from the primary auditory cortex (Ponton et al., 2002). In line with our findings, the N100/P200 complex is observed following a range of different sensory stimuli (Mouraux & Iannetti, 2009;Singhal et al., 2002), suggesting this ERP may reflect higher-level processing of perceptual inputs from a broad cortical network, including the anterior cingulate cortex, insula, and thalamus (Downar et al., 2002;Mouraux et al., 2011). The size and distribution of N100/P200 potentials make it possible that other TEPs were masked by the strong PEPs (Gordon et al., 2023b;Ross, Ozdemir, et al., 2022). We did not include a condition in which sensory experiences were completely suppressed, so we cannot rule out that transcranial stimulation of the cortex also evokes potentials that contribute to the N100/P200 complex for each of the sites studied. Both online and offline methods for minimising PEPs may help uncover any additional TEPs masked by PEPs.

In addition to a generalised sensory response, we also observed early latency TEPs (<60 ms) that were specific to the site of stimulation, with peaks largest in the electrodes near the stimulated cortical region. This finding replicates a growing number of studies that have reported site specificity of early TEPs (Casarotto et al., 2011;Fecchio et al., 2017;Kähkönen et al., 2004;Ozdemir et al., 2020;Passera et al., 2022;Rogasch et al., 2020;Rosanova et al., 2009). More specifically, we replicated TEP peaks including an N40 following dorsolateral prefrontal cortex stimulation (Eshel et al., 2020;Kerwin et al., 2018;Rogasch et al., 2015), a P20 and N45 following premotor cortex stimulation (Belardinelli et al., 2019;Casarotto et al., 2011,[Bibr b7][Bibr b68]), and a P20 and N35 following parietal cortex stimulation (Belardinelli et al., 2019;Casarotto et al., 2010;Rosanova et al., 2009). While the origin of these early TEPs is unclear, several lines of evidence suggest these peaks likely reflect neural activity generated by the site of stimulation, and reverberant activity throughout connected cortico-cortical and cortico-thalamic networks initiated by TMS. First, as observed in this study, site-specific early TEPs show weak correlations with PEPs from sensory control conditions and show spatial characteristics consistent with activity from the site of stimulation. Second, early TEPs are not altered by changing sensory experiences of TMS, like the level of TMS click perception. Third, early TEPs are only present when healthy cortical tissue is stimulated, not when lesioned tissue is stimulated (Gosseries et al., 2015). Finally, site-specific early TEPs with similar latencies and invariance to sensory input have also been observed from invasive cortical recordings in non-human primates (Perera et al., 2023). Together, these findings suggest that early latency TEPs most likely primarily reflect neural activity resulting from transcranial stimulation of the cortex, both at the site of stimulation and in broader networks.

Of note, we also found that residual TMS-evoked muscle activity distorted early TEPs in a subgroup of individuals despite cleaning with ICA. Distortion was particularly prominent following prefrontal cortex stimulation, where TMS-evoked muscle activity is larger and more common across individuals due to the lateral position of the cortical target, and the likely TMS-evoked neural activity underlies the source of artefact (i.e., the cranial and facial muscles). While the presence/absence of residual muscle activity did not fully explain the site specificity of early TEPs, the finding highlights a broader challenge in accurately uncovering neural activity from highly artefactual TMS-EEG signals, and the limitations of methods like ICA. Despite the popularity of ICA for cleaning TMS-EEG data, several studies have shown that the high amplitude and time-locked nature of artefacts like TMS-evoked scalp muscle activity violate the assumptions of ICA, and can lead to misleading outcomes (e.g., neural activity removed with artefactual signal or distortion of the remaining signal) (Atti et al., 2024;Hernandez-Pavon et al., 2012;Metsomaa et al., 2014). Several other approaches have been suggested for minimising TMS-evoked scalp muscle activity such as PCA, SOUND, and SSP-SIR (Hernandez-Pavon et al., 2022). However, it remains an open issue as to whether any of these approaches can accurately uncover early TEPs in the presence of TMS-evoked scalp muscle activity (Hernandez-Pavon et al., 2023).

## 5 Limitations

There are several important limitations to the current study. First, we used white noise played through in-ear headphones to mask the clicking sound of TMS. Currently, there are three main approaches for masking the TMS clicking sound: (1) playing white noise through headphones, (2) playing noise with a frequency spectrum adapted from the TMS click sound itself, or more recently (3) playing noise with a customised frequency spectrum using the TAAC toolbox (Russo et al., 2022). While certain studies have suggested that white noise and adapted noise are roughly equivalent in reducing the N100/P200 amplitude (Ter Braack et al., 2015), more recent studies have shown that customised noise is more effective at reducing the participants perception of the clicking sound than white or adapted noise masking (Russo et al., 2022). When considering TEPs, near complete suppression of the N100/P200 peaks during auditory sham TMS conditions has been reported when using adapted and customised noise masking for some stimulation sites (Massimini et al., 2005;Rocchi et al., 2021;Russo et al., 2022), with customised noise masking substantially reducing TMS-related auditory-evoked potentials in auditory brain regions measured using intracranial EEG in one individual (Trapp et al., 2024). The subjective ratings of click sound perception in our study suggested that white noise was not able to completely mask the TMS click sound or auditory-evoked potential for most individuals and stimulation sites, particularly following DLPFC stimulation. Similar to our findings, a recent study showed no reduction in the N100/P200 peak amplitude between no noise masking and adapted noise masking following DLPFC stimulation (Ross, Sarkar, et al., 2022), suggesting adapted noise masking may not be suitable for all sites either. It is possible that more effective suppression of the TMS click sound perception could have been achieved if customised (e.g., with the TAAC toolbox) noise masking was used instead of white noise in the current studies. Second, while the site-specific characteristics of the early latency TEPs (i.e., <60 ms) are consistent with activity evoked by transcranial stimulation of the cortex, we cannot rule out that sensory potentials also contribute in some way to these early peaks. Changing the level of auditory input did not alter early TEP amplitudes for any of the stimulation sites, making the contribution of auditory potentials unlikely. However, the contribution of somatosensory potentials is less clear. Recently, a sham condition was developed which experimentally saturates somatosensory input by using concurrent electrical stimulation of the scalp with TMS (Gordon et al., 2021). This approach was used to rule out somatosensory contributions to early TEPs following stimulation of motor cortex (Gordon et al., 2023b), dorsolateral prefrontal cortex, supplementary motor cortex (close to premotor cortex stimulation site in our study), and angular gyrus (close to parietal cortex stimulation site in our study) (Song et al., 2024). Together, these findings provide further evidence suggesting that early TEPs largely reflect activity resulting from transcranial stimulation of the cortex. Third, the sample size in Experiment C (n = 12) was lower than that in Experiments A and B. While the sample size appeared appropriate to detect changes in TEPs with differing levels of noise masking for some stimulation sites (e.g., parietal cortex), it is possible that the experiment was underpowered to detect small changes in TEPs from other sites (e.g., prefrontal and premotor cortex). Fourth, stimulation of the pre-supplementary motor area (close to the premotor site in the current study) can elicit MEPs at suprathreshold intensities, most likely due to co-stimulation of the primary motor cortex (e.g., the e-field extends to the hand region of the primary motor cortex following premotor stimulation; seeFig. 3A) (Casula et al., 2022). The corresponding muscle twitch results in reafferent sensory input to the cortex which can alter TEP amplitudes (Biabani et al., 2021;Fecchio et al., 2017;Petrichella et al., 2017). We did not record EMG during stimulation of the different cortical sites, so cannot rule out that reafferent sensory input contributed to TEP amplitudes following premotor stimulation, although this possibility is less likely in Experiments B and C which used lower stimulation intensities. Finally, we used ICA to minimise the contribution of several non-neural artefacts to TEPs in addition to TMS-evoked scalp muscle activity, including decay artefacts, ongoing scalp muscle activity, blinks, and eye movements. ICA can result in either undercleaning of the data if artefact signal is not completely removed or overcleaning if neural signal is removed alongside artefactual signal. As such, it is possible that differences in early TEPs between stimulation sites were driven by differences in artefact profiles and the subsequent ICA-based cleaning procedures required to minimise these artefacts, as opposed to representing TEPs from the stimulated cortical region. The optimal online and offline approaches for minimising artefact contributions to TEPs remain an open issue in TMS-EEG research (Hernandez-Pavon et al., 2023) and the current results should be interpreted with caution. Future work should use online feedback methods such as the rt-TEP toolbox (Casarotto et al., 2022) to fine-tune stimulus parameters and reduce artefacts (e.g., cranial muscle activation) during data acquisition to more accurately capture early TEP brain responses.

## 6 Implications

An important implication of the current findings is that both auditory and somatosensory input can induce potentials which contribute to TEPs. While it is possible that auditory input can be minimised across different sites with adequate noise masking, controlling the level of somatosensory input is much more challenging, especially for more lateral sites which experience higher levels of TMS-evoked muscle twitches like the DLPFC. Therefore, it may not be possible to completely eliminate the contribution of sensory potentials from TEPs using experimental arrangements for certain stimulation sites. As such, the use of well-designed experimental control conditions and the continued development of off-line approaches is required for assessing and minimising sensory potentials in TEPs (Hernandez-Pavon et al., 2023;Rogasch et al., 2022). Our findings also highlight the importance of considering sensory experiences when comparing TEPs between different stimulation sites and different groups which may have differences in sensory perception. For example, older individuals (Kok, 2000) and individuals with psychiatric (Javitt, 2009;Shen et al., 2020) and neurological (Horvath et al., 2018) conditions show differences in sensory-evoked potentials. Therefore, differences in TEPs between these groups could be attributed to either changes in sensory perception or the physiology of targeted neural circuits following TMS or a combination of the two. Careful experimental design is required to disentangle these possible contributions in between-group TMS-EEG studies.

## 7 Conclusions

The present findings suggest that frontocentral TEPs, particularly around the N100/P200 peaks, most likely primarily reflect sensory-evoked potentials if sensory experiences are reported following TMS as they (1) showed high spatial and temporal correlations between stimulation sites and with sensory control conditions; (2) differed in amplitude between individuals who experienced more versus less sensory input from TMS; and (3) differed in amplitude within individuals when the level of TMS click sound perception was modulated. Importantly, we also showed that later TEP amplitudes were larger following stimulation of sites with more sensory input like the DLPFC and were larger in individuals with higher levels of click sound and TMS-evoked scalp muscle twitch perception, suggesting both auditory and somatosensory inputs contribute to TEPs. In contrast, short-latency TEPs (~16–60 ms) most likely primarily reflect neural activity resulting from transcranial stimulation of the cortex as they were (1) lateralised to the site of stimulation, (2) uncorrelated with sensory-evoked potentials from sensory control conditions without active TMS, and (3) invariant to changes in the level of TMS click sound perception. However, the spatiotemporal characteristics of early TEPs were also distorted by residual artefacts in a subgroup of individuals following prefrontal cortex stimulation despite cleaning with ICA, highlighting the challenges in uncovering neural activity in the presence of TMS-evoked muscle artefacts. Our findings have important implications for the design and interpretation of TMS-EEG studies, suggesting sensory potentials may be more difficult to control experimentally for certain stimulation sites where muscle twitches are unavoidable. Furthermore, our findings highlight the importance of accounting for sensory potentials when comparing TEPs between stimulation sites and participant cohorts with different levels of sensory perception.

## Supplementary Material

Supplementary Material

## Data Availability

Processed TMS-EEG data from Experiments A–C are available athttps://doi.org/10.26180/24514741. Analysis code is available athttps://github.com/ManaBiabani/NonMotor_TEPs_PEPs.git.
