# Supplementary material for: Characterising the contribution of auditory and somatosensory inputs to TMS-evoked potentials following stimulation of prefrontal, premotor, and parietal cortex
Source: Imaging Neurosci (Camb). 2024 Nov 1;2:imag-2-00349. doi: 10.1162/imag_a_00349 (PMC12290563; doi:10.1162/imag_a_00349)
Supplement: Supplementary Material [file imag_a_00349-supp.pdf]

Table S1 Mean±SD of stimulation parameters (Experiment B)

	Distance (mm)	Intensity (%MSO)
**Prefrontal**	18.15±3.83	56.59±12.12
**Premotor**	21.30±4.57	62.02±16.94
**Parietal**	20.02±4.53	61.67±12.59
**Shoulder**	–	52.77±10.73

Distance: scalp-to-cortex distance; Intensity: Distance-adjusted stimulation intensity; %MSO: Percentage of maximum stimulator output.

Table S2 Mean±SD of stimulation parameters (Experiment C)

	Distance (mm)	TMS Intensity (%MSO)	ES Intensity (mA)
**Prefrontal**	18.71±4.44	38.5±11.02	0.65±0.16
**Premotor**	21.4±6.68	44.42±7.64	0.73±0.31
**Parietal**	23.01±6.18	46.92±8.22	0.73±0.30
**Shoulder**	–	45.52±10.83	–

Distance: scalp-to-cortex distance; Intensity: Distance-adjusted stimulation intensity; %MSO: Percentage of maximum stimulator output.

Table S3. Mean±SD of the number rejected components and the variance they explained

	Experiment A		Experiment B		Experiment C	
	**Count**	**Variance**	**Count**	**Variance**	**Count**	**Variance**
**TMS-Evoked Muscle Activity**	3.91 ± 2.06	7.45 ± 13.26	2.96 ± 1.73	4.58 ± 12.34	1.12 ± 2.84	5.26 ± 11.75
**Muscle Contraction**	6.65 ± 4.90	1.36 ± 1.76	11.43 ± 7.07	3.57 ± 11.28	4.16 ± 2.62	1.01 ± 1.53
**Electrode Noise**	19.68 ± 3.45	7.01 ± 6.40	17.81 ± 6.72	5.87 ± 11.31	15.41 ± 3.44	5.11 ± 3.52
**Blink**	2.65 ± 1.47	57.80 ± 20.19	2.95 ± 1.81	66.51 ± 23.58	2.66 ± 1.82	54.68 ± 23.88
**Eye Movement**	0.65 ± 0.48	0.93 ± 1.22	0.71 ± 0.52	0.46 ± 0.61	1.01 ± 0.73	2.71 ± 6.77

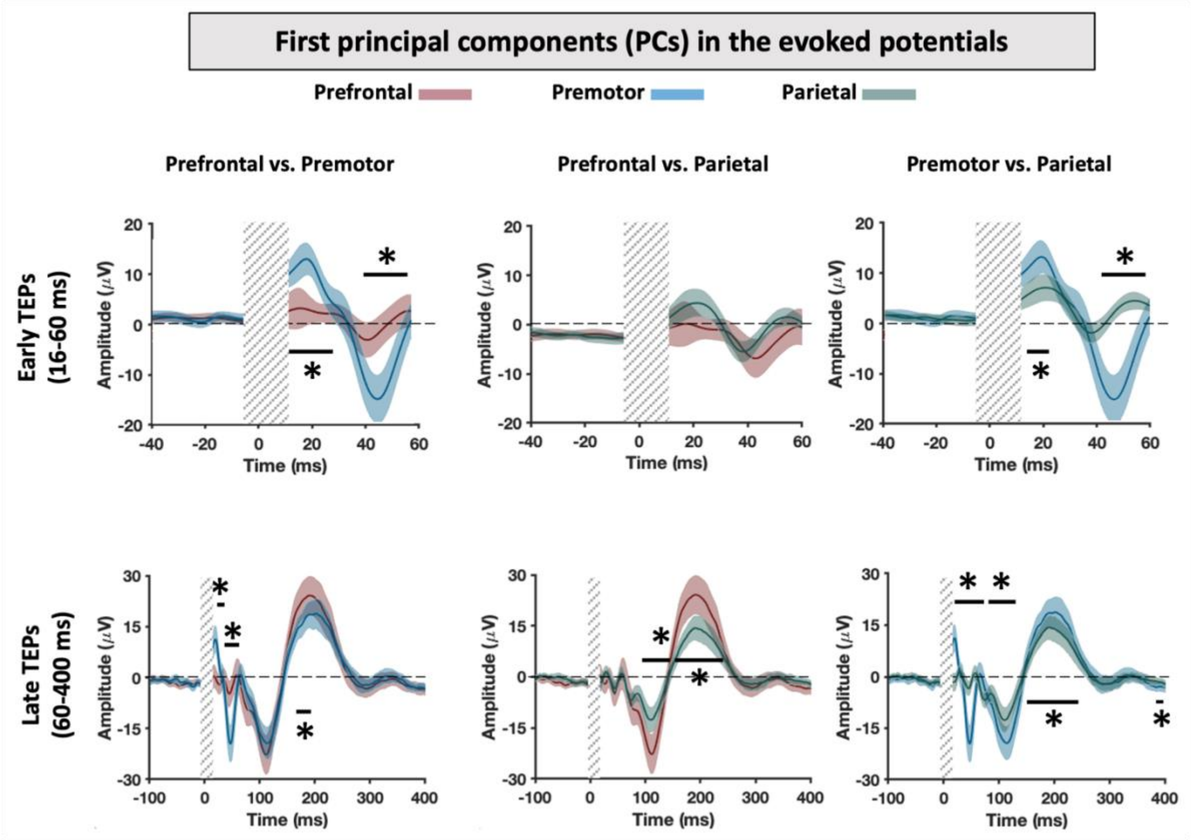

Figure S1. Comparison of TEP first principal components (PCs) between stimulation sites. Top) Time series of the dominant PCs identified in early TEPs recorded between 16 and 60ms. Bottom) Time series of the dominant PCs identified in late TEPs recorded between 60 and 400ms. The line graphs illustrate the changes of PCs amplitude over time. The thick lines represent the group averaged signal and the shaded areas show 95% CIs of the individual values. The vertical grey bars demonstrate the time-window of the potentials not considered for the analysis. The horizontal lines with * indicate when the TEPs significantly deviate between stimulation sites (corrected $p < 0.05$).

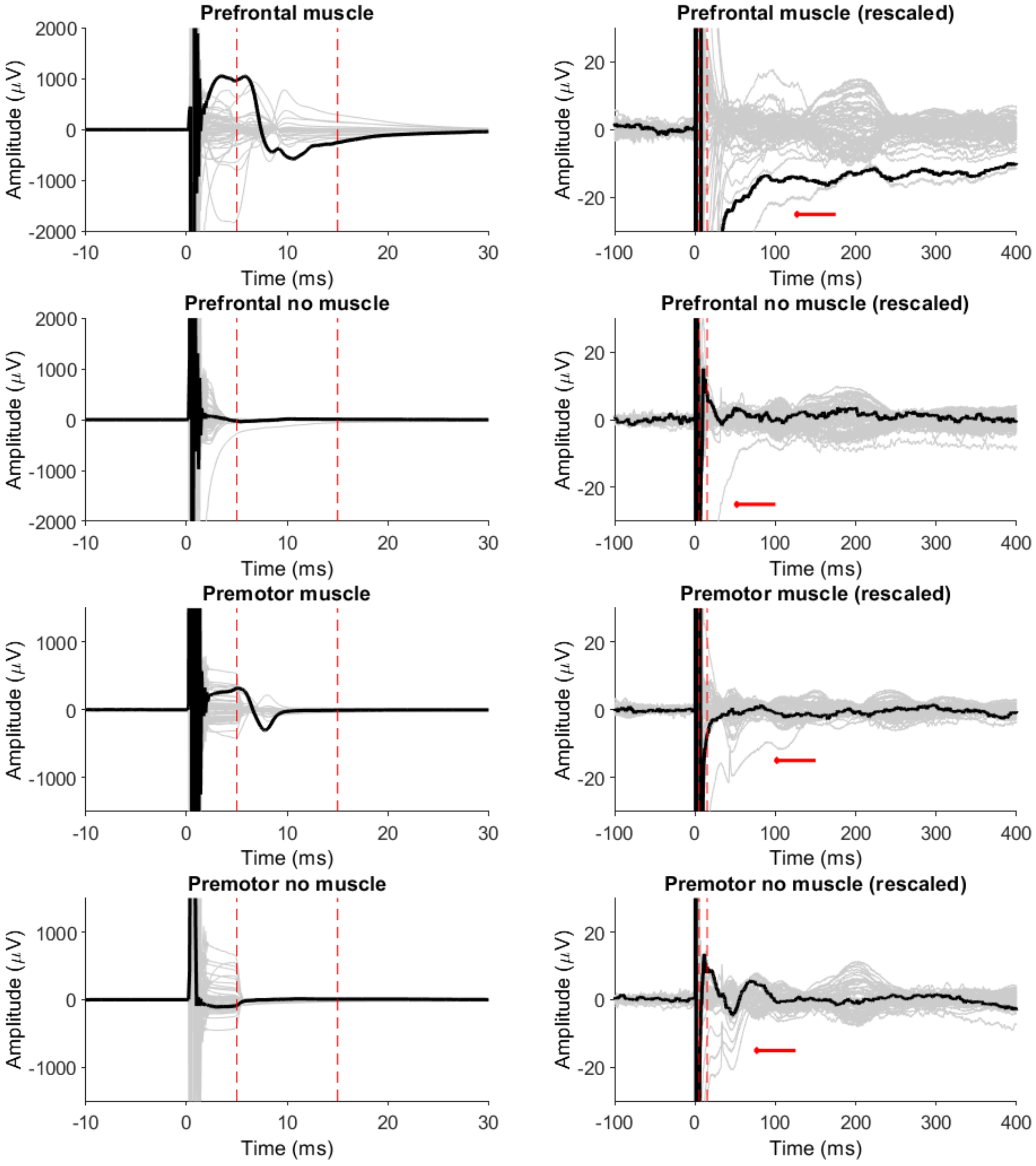

Figure S2. The method used to identify TMS-evoked scalp muscle activity in uncleaned TEP data. The presence/absence of a TMS-evoked scalp muscle artifact for a given condition was determined by visually assessing uncleaned TEPs. To generate uncleaned TEPs, the raw data were epoched around the TMS pulse, baseline corrected (-500 to -10 ms), disconnected electrodes were removed and interpolated, the data were average referenced, and then averaged across trials. TEPs from all electrodes (lateral electrode F5 highlighted in black) were inspected between -10 to 30 ms, and a muscle artifact was deemed present if peaks which deviated from the background decay artifacts (i.e., decay artifacts resulting from charges stored at the skin-electrode interface) were evident between 5-15 ms (time period indicated by red dotted lines). TMS-evoked scalp muscle artifacts have been observed with biphasic peaks at latencies of ~4-6 ms and ~8-12 ms (Rogasch et al. 2013, Mutanen et al, 2013). We chose to begin the window at 5 ms as in some participants, the amplifier was offline for up to 5 ms post TMS (indicated by flat lines offset from 0 – see ‘premotor muscle’ and ‘premotor no muscle’ for examples). Furthermore, ringing artifacts from the TMS pulse artifact are present for up to 5 ms at the

sampling rates used in this study (Veniero et al, 2009, Rogasch et al, 2013). We note that this experimental arrangement precludes us from detecting scalp muscle artifacts within the first few ms following TMS which are theoretically possible, although we are not aware of any reports of such early scalp muscle artifacts in the literature. In the left column, examples are provided of individuals showing both presence and absence of TMS-evoked muscle activity in uncleaned TEPs following prefrontal and premotor cortex stimulation using the visualisation method. In the right column, rescaled figures of the uncleaned TEPs are provided which show the presence of decay artifacts related to charges stored at the skin-electrode interface in all conditions regardless of the presence/absence of TMS-evoke scalp muscle activity (indicated by red arrows). Note a sharp, high frequency artifact of unknown origin is present between 30-50 ms in some conditions. Capacitor recharge was delayed to 1000 ms post TMS for all conditions meaning this source of artifact is unlikely the cause, but might instead relate to some other componentry within the TMS device. This artifact was no longer evident following down sampling of the data.

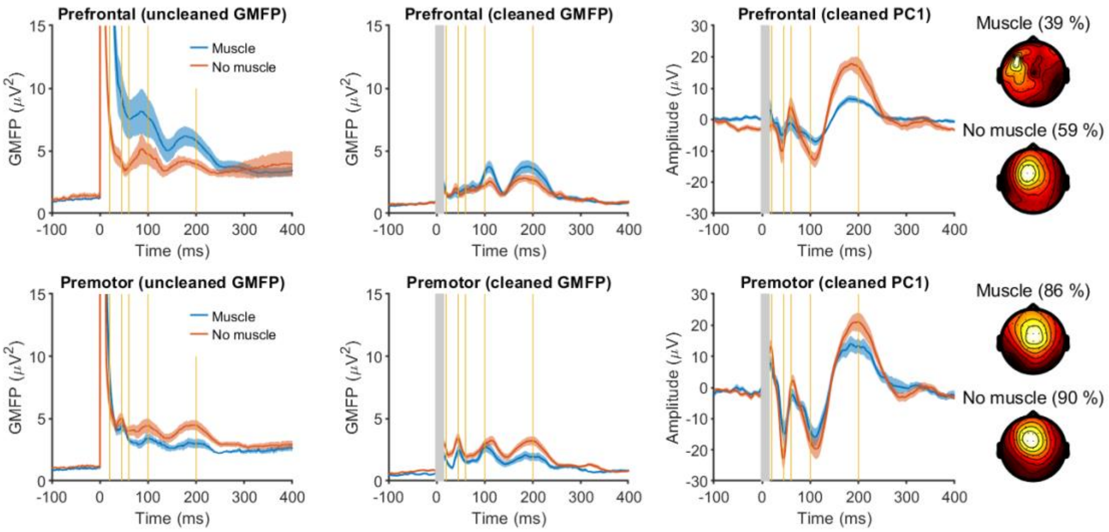

Figure S3. TEPs following prefrontal and premotor cortex stimulation split based on the presence of TMS-evoked scalp muscle activity visible in the uncleaned EEG trace. Left column: uncleaned global mean field potentials (GMFP) in individuals with (blue) and without (red) TMS-evoked scalp muscle activity within the first 15 ms. Note that the tail of the muscle activity continues to impact TEPs for >100 ms following prefrontal cortex stimulation, but not premotor stimulation. Also note that decay artifacts resulting from charges stored at the electrode-scalp interface which are independent from TMS-evoked scalp muscle activity are evident in all conditions. Middle column: GMFP of TEPs with and without TMS-evoked scalp muscle activity after cleaning (i.e., TMS pulse removal, downsampling, filtering and two rounds of ICA). Right column: the first principal component (PC1) of cleaned TEPs with and without scalp muscle activity, including the time series and the topographies (i.e., weightings across electrodes). Variance accounted for by each PC is shown in brackets above the topoplots. Note that PC1 following prefrontal cortex stimulation (with muscle) appears to still represent residual muscle activity despite cleaning with independent component analysis. This is not the case for individuals without scalp muscle activity following prefrontal stimulation, or in either group following premotor stimulation. Data are mean +/- standard error (shaded bars). The timing of the main peaks are indicated with yellow vertical lines. Topoplots are scaled from minimum to maximum values.

Figure S4 illustrates the levels of perception of pulses across the stimulated regions in experiments B. According to Shapiro-Wilk normality tests, NRS values were not normally distributed in all conditions (p>0.05). Therefore, we used Friedman tests for comparisons across all conditions, followed by FDR corrected Wilcoxon Signed-Rank tests for pairwise comparisons. Despite the differences in stimulation intensities, participants in experiment B showed almost the same pattern of perception across cortical sites as observed in experiment A; with prefrontal and parietal causing the strongest and weakest sensations, respectively. All sensations showed significant differences across stimulation sites (For pain Chi-square = 9.21, df = 3, p = 0.003; Pairwise FDR-Corrected p : prefrontal-premotor = 0.07, prefrontal-Parietal = 0.03, prefrontal-control = 0.03, premotor-Parietal = 0.31, premotor-control = 0.37; Parietal-control = 0.76; For discomfort Chi-square = 39.44, df = 3, p <0.0001; Pairwise FDR-Corrected p : prefrontal-premotor = 0.0005, prefrontal-Parietal <0.0001, prefrontal-control<0.0001, premotor-Parietal = 0.04, premotor-control = 0.16; Parietal-control = 0.92; For twitch Chi-square = 28.73, df = 3, p <0.0001; Pairwise FDR-Corrected p : prefrontal-premotor <0.0001, prefrontal-Parietal <0.0001, prefrontal-control = 0.0003, premotor-Parietal = 0.04, premotor-control = 0.24 and Parietal-control = 0.009; For sound Chi-square = 43.99, df = 3, p <0.0001; Pairwise FDR-Corrected p : prefrontal-premotor = 0.04, prefrontal-Parietal <0.0001, prefrontal-control<0.0001, premotor-Parietal = 0.0005, premotor-control = 0.001, Parietal-control = 0.34) (Figure S2).

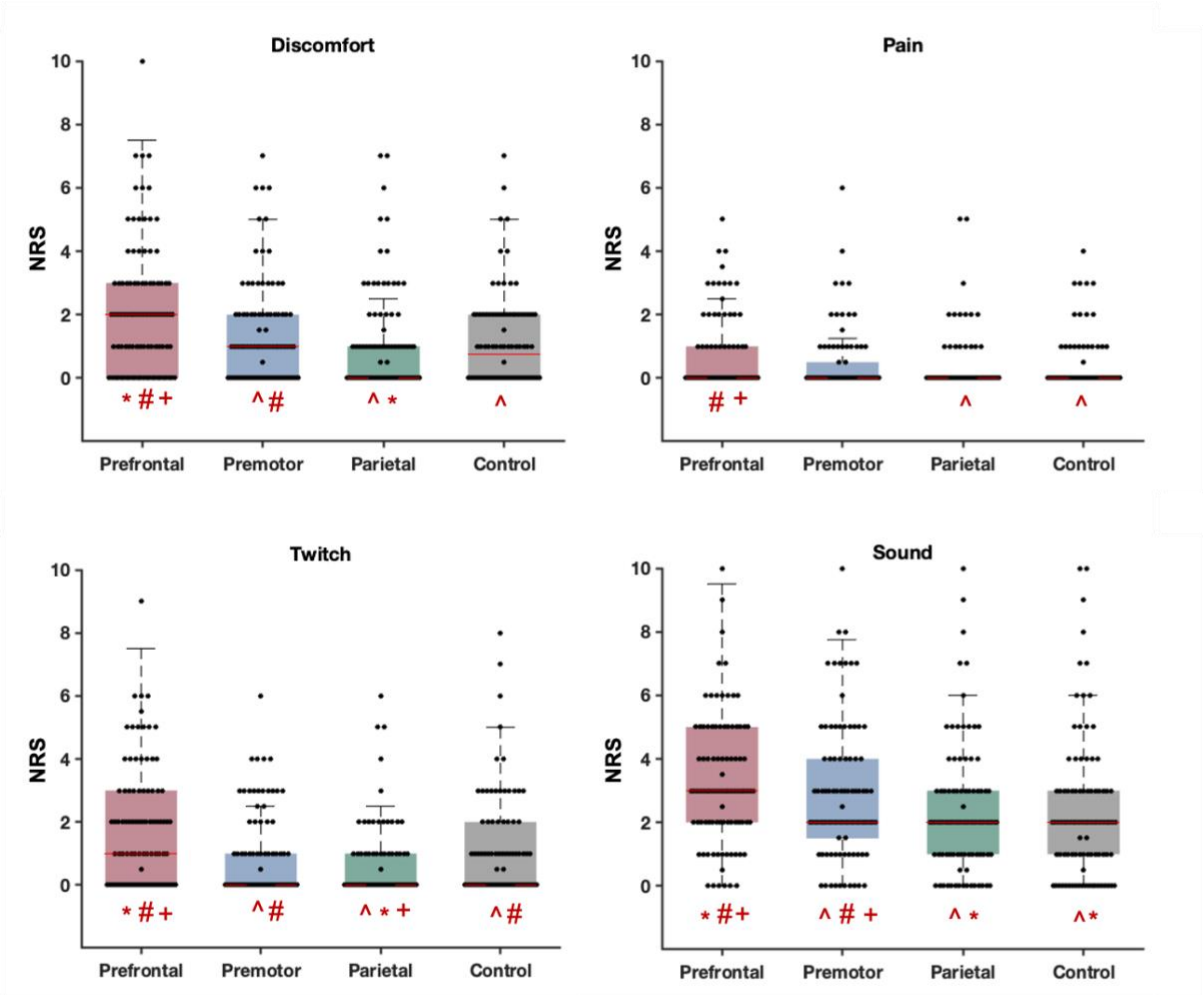

Figure S4. Experiment B- Self-reported perception of discomfort, pain, muscle twitch and click sound caused by real and control stimulation conditions. Each dot in the box and whisker plots represents the Numerical Rating Scale (NRS) score for each individual. The shaded boxes highlight the 25th to 75th centiles of the values and the red horizontal lines within the boxes show the median of the values. ^, *****, **#,** and **+** indicate significant difference (FDR-corrected $p<0.05$) with prefrontal, premotor, Parietal and control, respectively.

After the data cleaning process for Experiment B, the number of remaining trials (mean ± SD) in different conditions were as follows: prefrontal: 96.37 (16.68), premotor: 100.05 (14.61), parietal: 99.75 (18.72), and control: 101.06 (19.74).

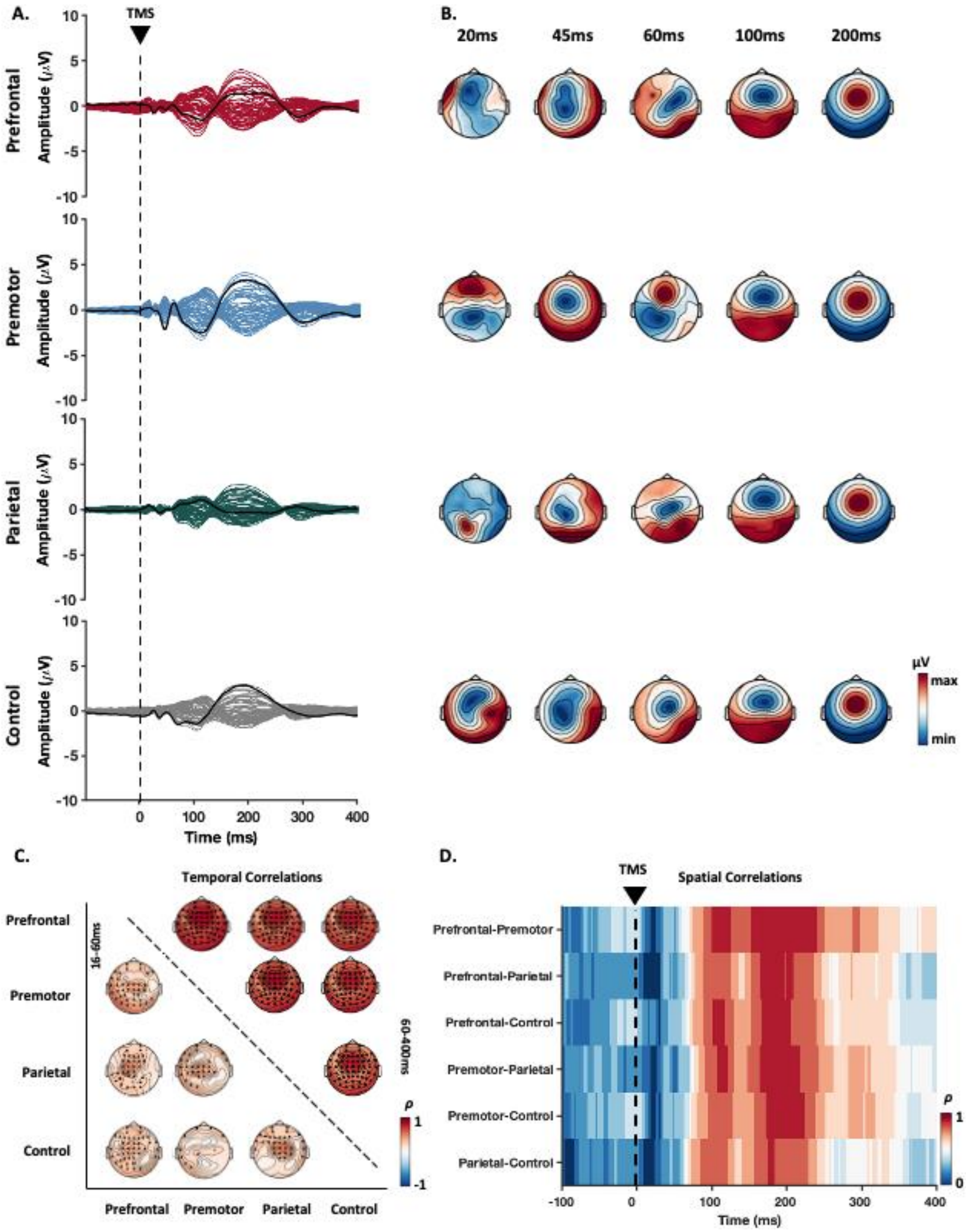

Figure S5. Experiment B- using subthreshold TMS: Spatiotemporal distribution of TEPs evoked by TMS over four different areas and their correlation among conditions.

A) Butterfly plots indicate TEPs recorded by each electrode averaged across individuals and the thick black line represent the potentials recorded at the targeted site (prefrontal: F3;

premotor: FC1, Parietal: P3 and Shoulder: CZ). B) Topographical maps depict distribution of the potentials across scalp around the timepoints that TEP peaks appeared. C) Spatial distribution of the Spearman's correlation coefficients (ρ) between the potentials recorded by the same electrodes in each two conditions at two different time windows; early (16-60ms; lower triangle) and late (60-400ms; upper triangle). The channels highlighted in black demonstrated significant correlations ($p_{corrected}<0.05$) between the two conditions. D) Temporal changes in Spearman's correlation between the topographies of each two conditions.

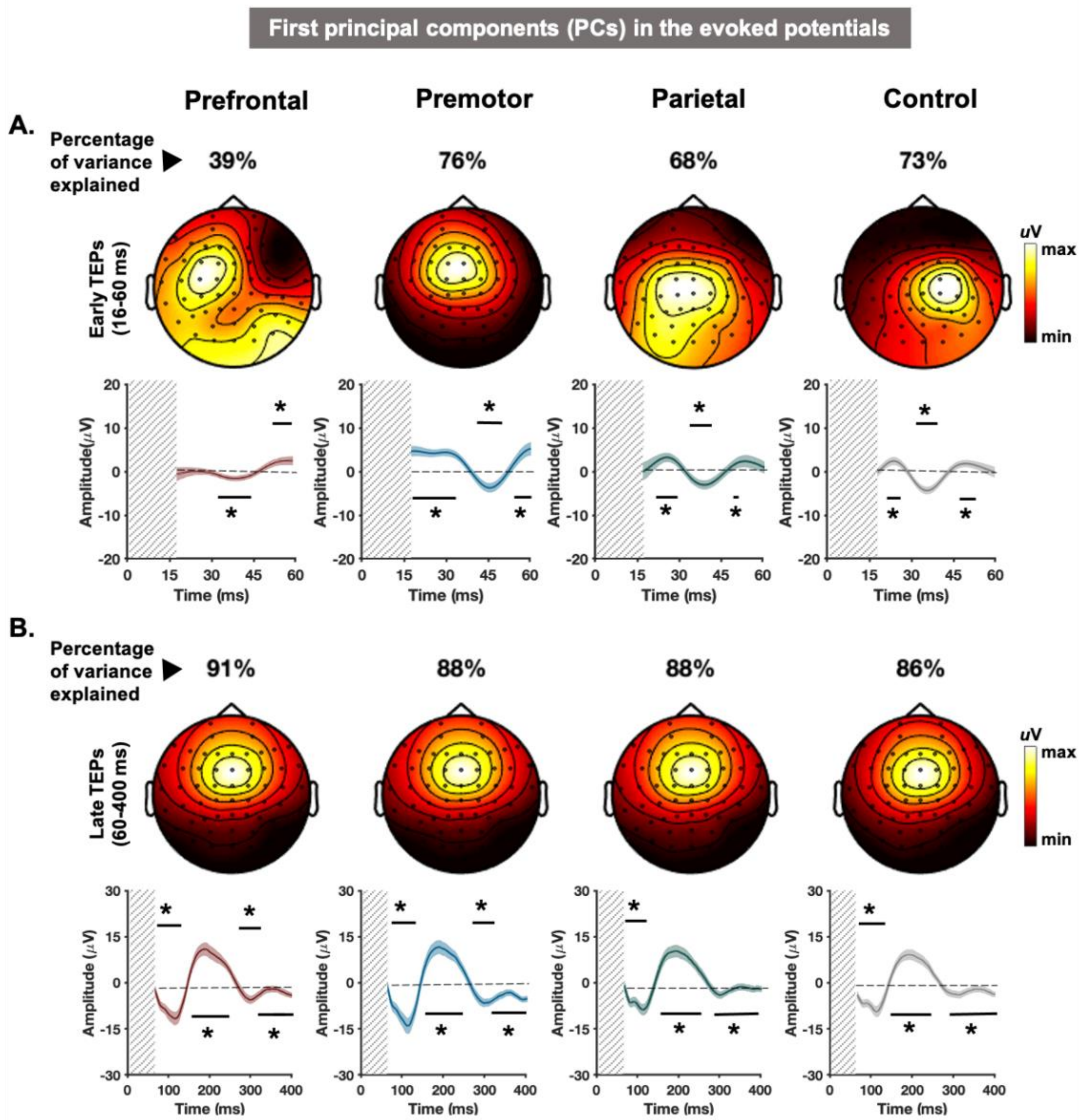

Figure S6. Experiment B- The principal components explaining the maximum variance of the potentials recorded at each stimulation condition. A) The dominant PCs identified in early TEPs recorded between 16 and 60ms. B) The dominant PCs identified in late TEPs recorded between 60 and 400ms. The values above the scalp maps indicate the percentage of variance explained by the depicted component for each condition. The line graphs illustrate the changes of PCs amplitude over time. The thick lines represent the group averaged signal and the shaded areas show 95% CIs of the individual values. The vertical grey bars demonstrate the time-window of the potentials not considered for the analysis. The horizontal lines with * indicate when the signals significantly deviate from baseline (corrected $p<0.05$).

After the data cleaning process for Experiment C, the number of remaining trials (mean ± SD) in different conditions were as follows for conditions with noise masking- prefrontal: 84.75 (9.65), premotor: 82.41(12.07), parietal: 76.58 (13.62), and shoulder: 80.58 (26.83); for conditions without noise masking- prefrontal: 83.16 (11.20), premotor: 83.01 (8.61), parietal: 84.91 (10.39) and for electrical stimulation- prefrontal: 84.01 (12.55), premotor: 90.08 (3.67), parietal: 85.33 (12.62).

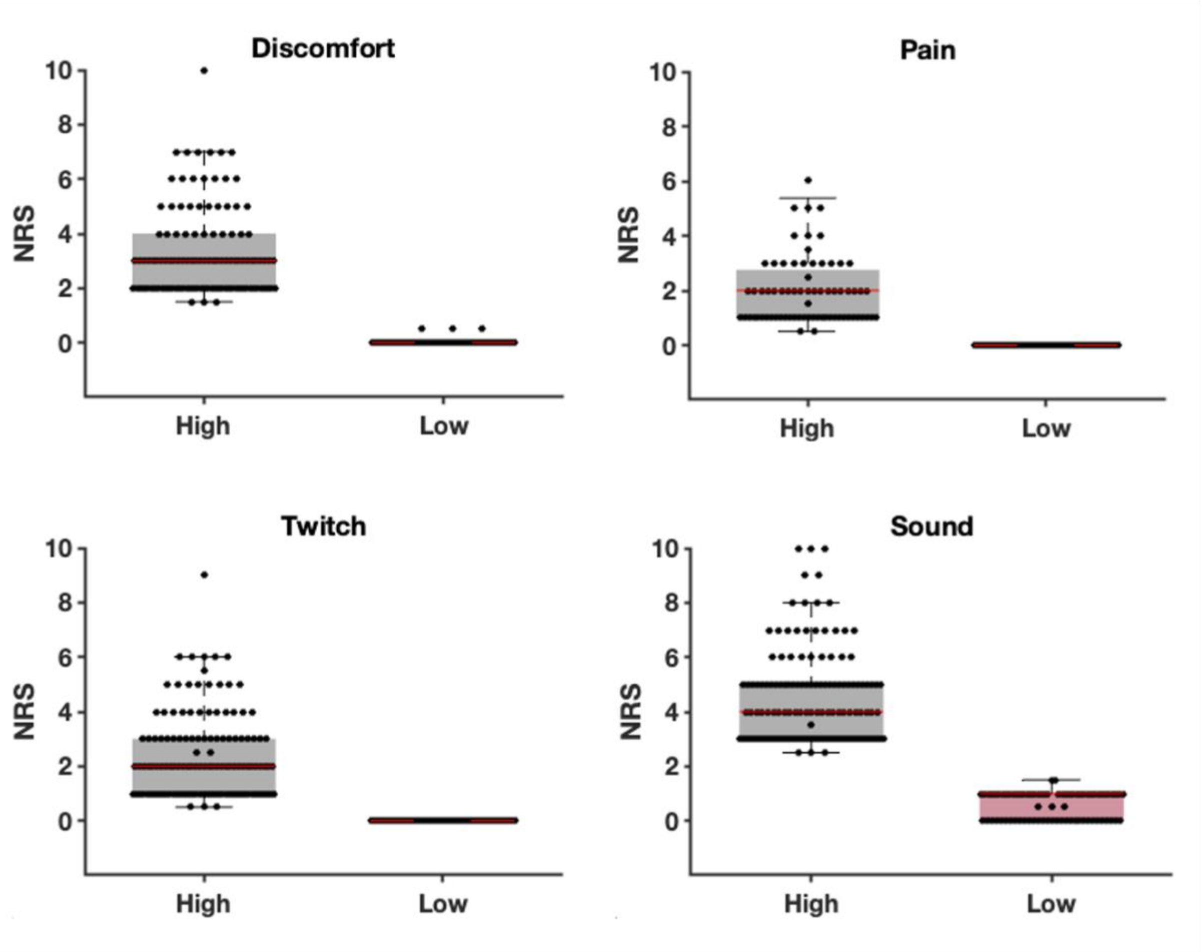

Figure S7. Experiment B- Distribution of self-reported perception of discomfort, pain, muscle twitch, and click sound following stratification of individuals into low (< median) and high (> median) levels of sensory perceptions. Values from all scalp stimulation conditions are pooled into each group. Each dot in the box and whisker plots represents the Numerical Rating Scale (NRS) score for each individual. The shaded boxes highlight the 25th to 75th centiles of the values and the red horizontal lines within the boxes show the median of the values.

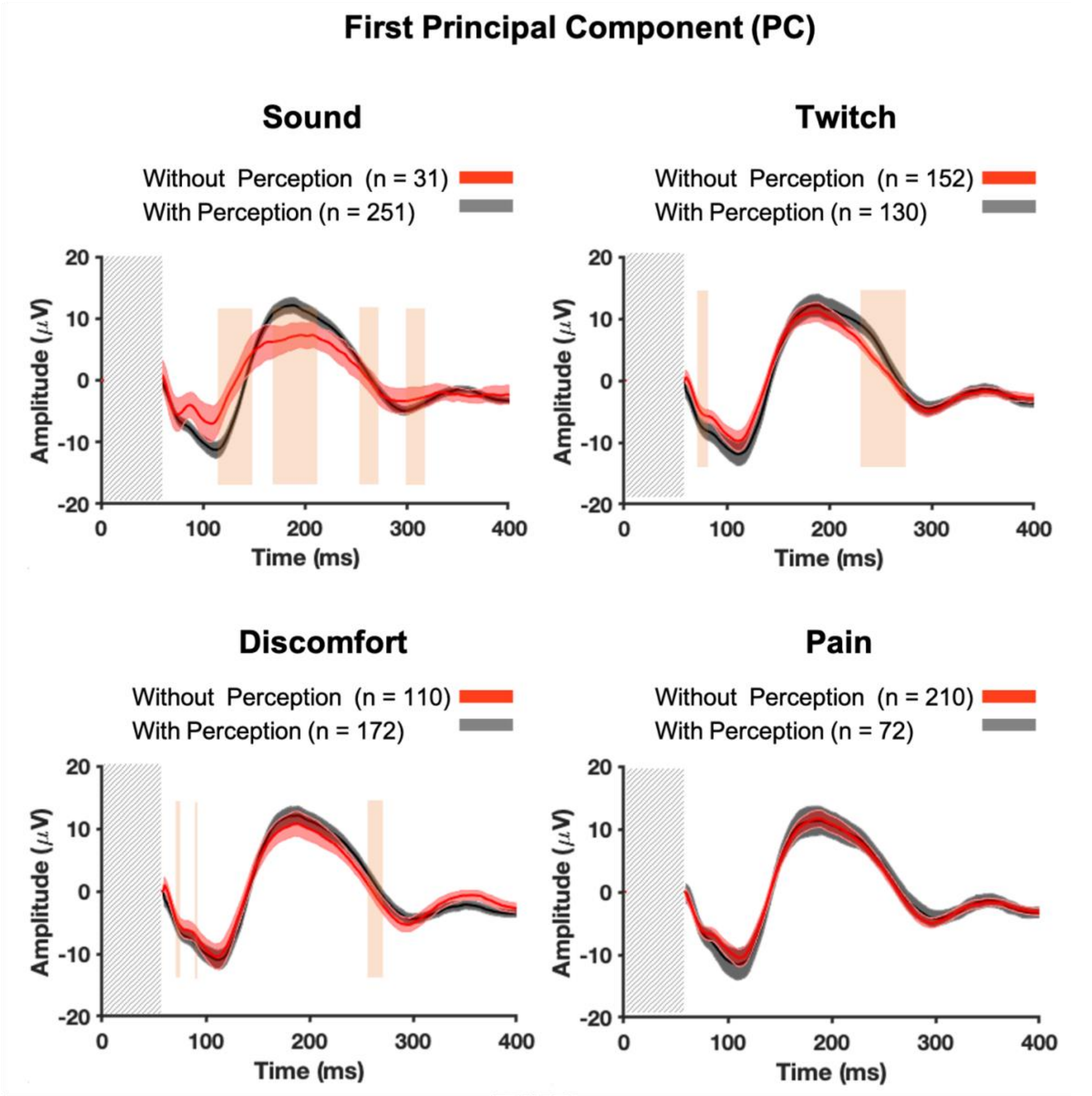

Figure S8. Experiment B- Comparisons of the late potentials between the individuals with high and low levels of sensory perceptions. A) Differences in the first PCs between the individuals with (NRS > 0) and without (NRS = 0) perception of TMS pulse. Potentials from all stimulation conditions are pooled into each group. The thick lines represent the group averaged signal and the shaded areas show 95% CIs of the individual values. The vertical grey bars demonstrate the time-window of the potentials not considered for the analysis. The orange vertical boxes cover the windows of time showing significant differences between groups (FDR-corrected P<0.05).

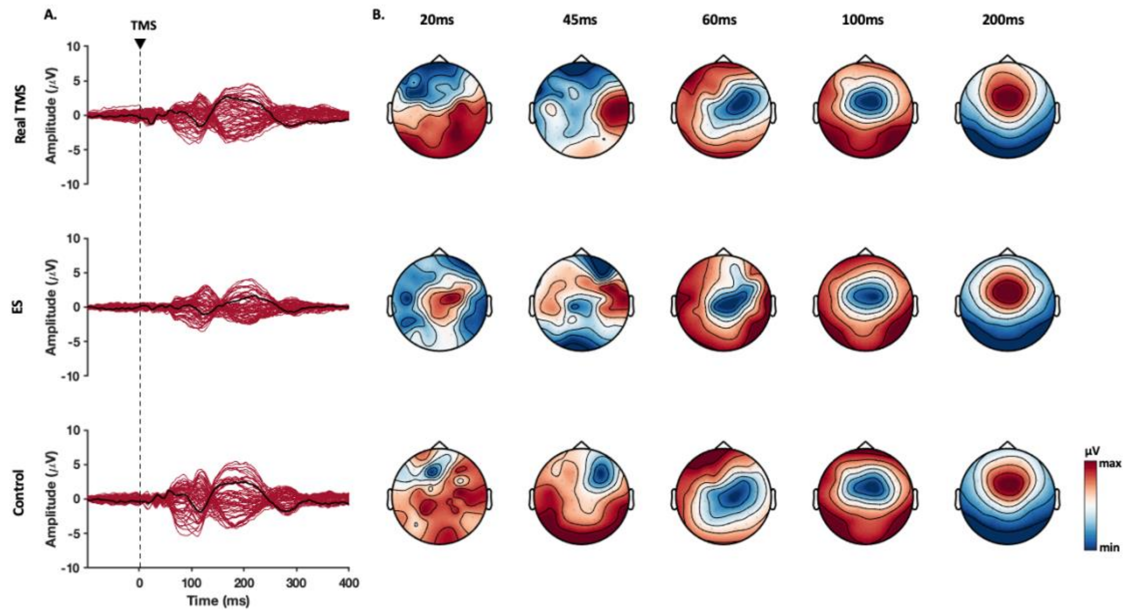

Figure S9. Experiment C- Spatiotemporal distribution of scalp recorded EEG potentials evoked by different stimulation conditions over the prefrontal cortex. In real TMS condition, stimulation was applied over the targeted area while the standard noise masking procedure was adopted. In the ES condition, electrical stimulation was applied over the targeted area, TMS coil was placed over the ES electrode tilted at 90° and the standard noise masking procedure was adopted. In control condition, TMS was applied without noise masking. B) Topographical maps depict distribution of the potentials across the scalp.

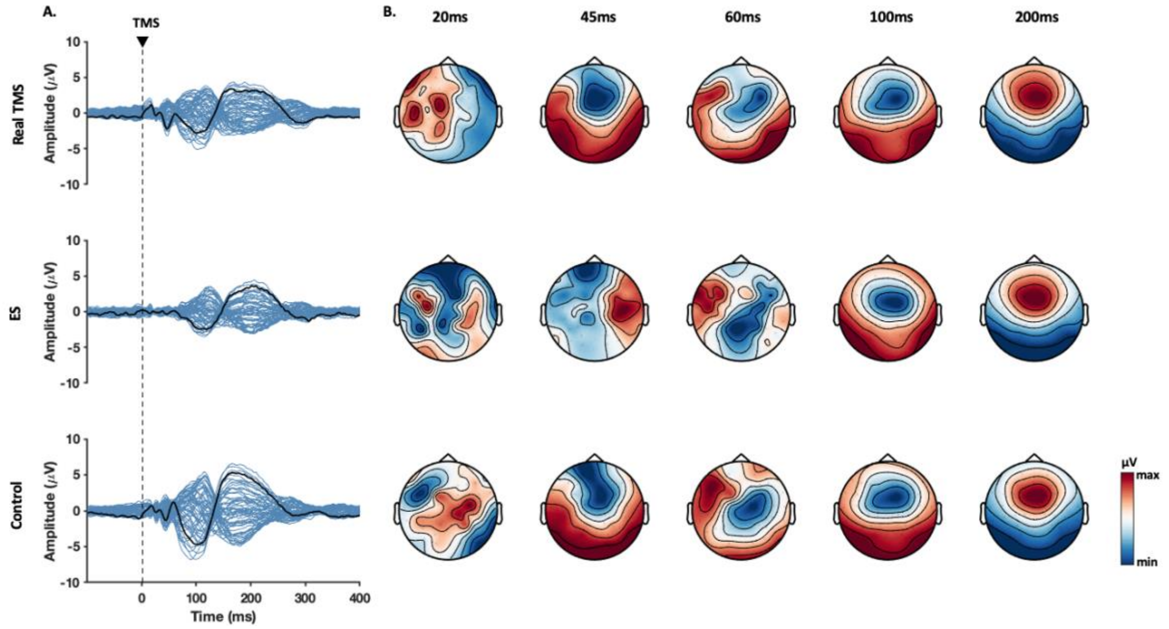

Figure S10. Experiment C- Spatiotemporal distribution of scalp recorded EEG potentials evoked by different stimulation conditions over the premotor cortex. In real TMS condition, stimulation was applied over the targeted area while the standard noise masking procedure was adopted. In the ES condition, electrical stimulation was applied over the targeted area, TMS coil was placed over the ES electrode tilted at 90° and the standard noise masking procedure was adopted. In control condition, TMS was applied without noise masking. B) Topographical maps depict distribution of the potentials across the scalp.

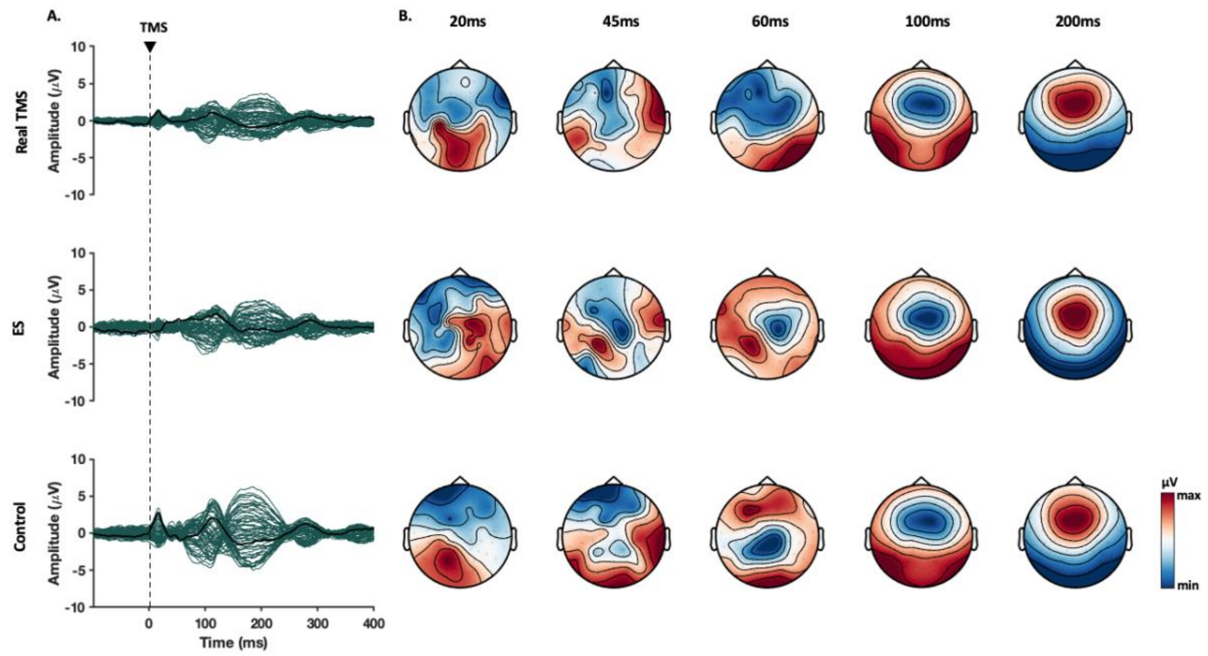

Figure S11. Experiment C- Spatiotemporal distribution of scalp recorded EEG potentials evoked by different stimulation conditions over the parietal cortex. In real TMS condition, stimulation was applied over the targeted area while the standard noise masking procedure was adopted. In the ES condition, electrical stimulation was applied over the targeted area, TMS coil was placed over the ES electrode tilted at 90° and the standard noise masking procedure was adopted. In control condition, TMS was applied without noise masking. B) Topographical maps depict distribution of the potentials across the scalp.

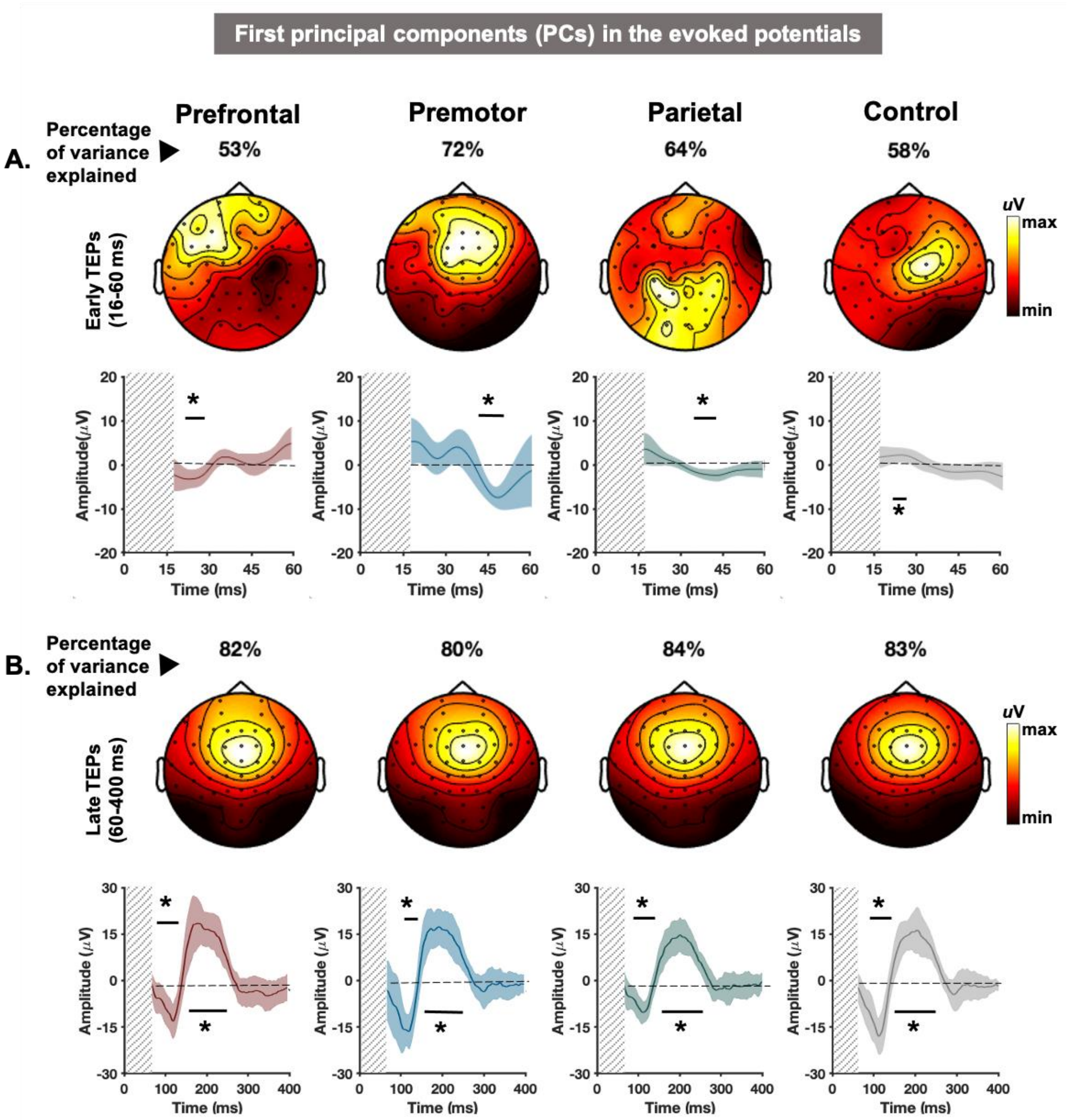

Figure S12. The principal components that explain the maximum variance of the potentials recorded at four different stimulation conditions in experiment C. The control condition represents TEPs from shoulder stimulation. A) The dominant PCs identified in early TEPs recorded between 16 and 60ms. B) The dominant PCs identified in late TEPs recorded between 60 and 400ms. The values above the scalp maps indicate the percentage of variance explained by the depicted component for each condition. The line graphs illustrate the changes of PCs amplitude over time. The thick lines represent the group averaged signal and the shaded areas show 95% CIs of the individual values. The vertical grey bars demonstrate the time-window of the potentials not considered for the analysis. The horizontal lines with * indicate when the signals significantly deviate from baseline (corrected $p<0.05$).

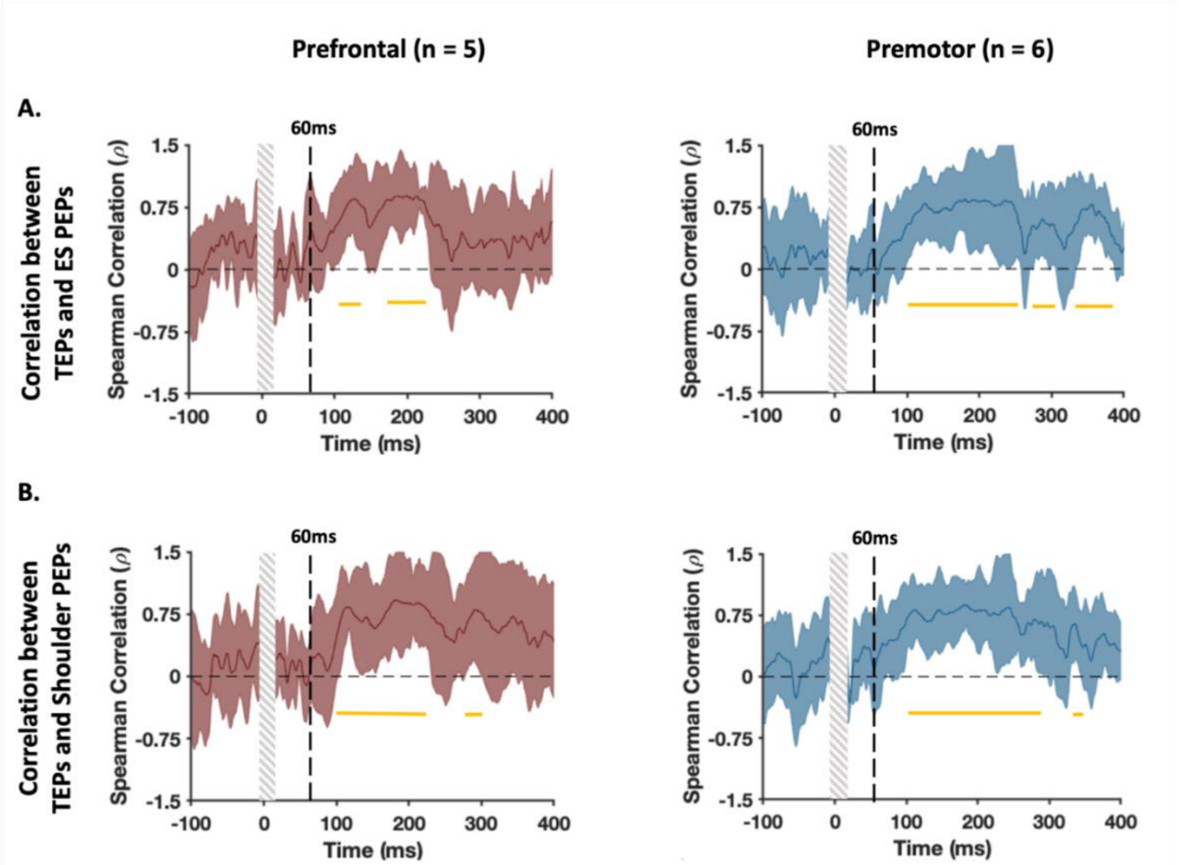

Figure S13. Sensory perceptions and TEPs from real TMS and somatosensory control (electrical stimulation) conditions in individuals without TMS-evoked scalp muscle activity. A-B) Changes in correlations between the GMFP of the responses to real and sham stimulations over time. The horizontal lines below the zero line indicate the windows of significant correlations. The thick line represents the group-averaged values. The shaded areas show 95% CIs of the values and the vertical grey bars demonstrate the time-window that was not considered for the analysis.

References

Mutanen, T., Mäki, H., & Ilmoniemi, R. J. (2013). The effect of stimulus parameters on TMS-EEG muscle artifacts. Brain Stimulation, 6(3), 371–376. https://doi.org/10.1016/j.brs.2012.07.005

Rogasch, N. C., Thomson, R. H., Daskalakis, Z. J., & Fitzgerald, P. B. (2013). Short-latency artifacts associated with concurrent TMS-EEG. Brain Stimulation, 6(6), 868–876. https://doi.org/10.1016/j.brs.2013.04.004

Veniero, D., Bortoletto, M., & Miniussi, C. (2009). TMS-EEG co-registration: On TMS-induced artifact. Clinical Neurophysiology: Official Journal of the International Federation of Clinical Neurophysiology, 120(7), 1392–1399. https://doi.org/10.1016/j.clinph.2009.04.023
